# Inhibition of AMPKα Pathway by Podocyte GOLM1 Exacerbates Diabetic Nephrology in Mice

**DOI:** 10.1002/advs.202505695

**Published:** 2025-07-13

**Authors:** Peng Xu, Kaiqiang Li, Huasong Liu, Aibo Xu, Zhewei Zhang, Yimin Yang, Xiaobo Lai, Ke Hao, Kui Fang, Zeyu Lai, Xiping Ou, Yuqun Cai, Zhen Wang, Keda Lu, Wanli Jiang

**Affiliations:** ^1^ The Third Affiliated Hospital of Zhejiang Chinese Medical University Hangzhou Zhejiang 310009 China; ^2^ School of Medical Technology and Information Engineering Zhejiang Chinese Medical University Hangzhou Zhejiang 310009 China; ^3^ Laboratory Medicine Center Allergy Center Department of Transfusion Medicine Zhejiang Provincial People's Hospital Affiliated People's Hospital Hangzhou Medical College Hangzhou Zhejiang 310014 China; ^4^ Department of Thoracic Surgery Renmin Hospital of Wuhan University Wuhan Hubei 430060 China

**Keywords:** AMPK, diabetic nephrology, fibrosis, inflammatory response, oxidative damage, podocytes

## Abstract

Inflammation and oxidative stress contribute to diabetic nephrology (DN) progression. Golgi membrane protein 1 (GOLM1) is a Golgi type II transmembrane protein and associates with inflammation and oxidative stress. The present study tries to investigate the role and mechanism of GOLM1 in DN using gain‐ and loss‐of‐function approaches. It is found that GOLM1 expression is elevated in diabetic kidneys and high glucose‐stimulated podocytes, and positively correlated with renal dysfunction during DN progression. Podocyte‐specific GOLM1 ablation prevents, while podocyte‐specific GOLM1 overexpression facilitates diabetes‐related inflammation, oxidative damage, apoptosis, and renal dysfunction in vivo and in vitro. Mechanistic studies reveal that podocyte‐specific GOLM1 ablation attenuated DN through activating adenosine monophosphate activated protein kinase α (AMPKα) pathway, and inhibiting AMPKα pathway completely abolishes the beneficial effects in podocyte‐specific GOLM1 knockout (*Golm1* ^pKO^) mice or podocytes. Further findings imply that podocyte GOLM1 inactivated AMPKα pathway through interacting with epidermal growth factor receptor to inhibit peroxisome proliferator activated receptor γ. Moreover, treatment with GOLM1 neutralizing antibody is sufficient to alleviate DN in mice. Overall, the study for the first time identifies a pathogenic role of podocyte GOLM1 in the progression of DN, and inhibiting GOLM1 represents an attractive therapeutic approach to treat DN.

## Graphical abstract

Podocyte Golgi membrane protein 1 interacts with epidermal growth factor receptor to inhibit peroxisome proliferator activated receptor γ, and then inactivates adenosine monophosphate activated protein kinase α pathway, which facilitates diabetes‐related inflammation, oxidative damage, apoptosis, and renal dysfunction.

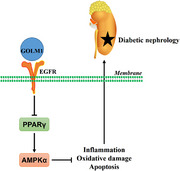

## 1 Introduction

Diabetic nephrology (DN) is a major microvascular complication in diabetic patients that results in end‐stage renal disease and high mortality worldwide.^[^ ^1^, ^2^, ^3^ ^]^ Multiple mechanisms contribute to the progression of DN, including inflammation and oxidative stress.^[^ ^4^, ^5^ ^]^ Guo et al. recently found that nuclear factor‐κB (NF‐κB) was activated in the kidneys of diabetic mice, accompanied with a significantly elevated release of proinflammatory cytokines, and that inhibiting inflammation effectively prevented diabetes‐induced renal dysfunction.^[^ ^6^ ^]^ In contrast, increasing inflammation facilitated fat deposition and glomerular sclerosis, thereby fueling DN progression.^[^ ^7^ ^]^ Meanwhile, reactive oxygen species (ROS) overproduction is the other feature and pathogenic factor of DN. Yang et al. previously demonstrated that the redox enzyme p66Shc was upregulated in diabetic kidneys and subsequently aggravated oxidative stress during DN progression.^[^ ^8^ ^]^ And inhibiting oxidative damage could alleviate DN in mice.^[^ ^9^ ^]^ Therefore, finding negative regulators of inflammation and oxidative stress is of great therapeutic interest to treat DN.

Adenosine monophosphate activated protein kinase α (AMPKα), a major energy sensor, plays critical roles in the regulation of cellular energy homeostasis; however, emerging studies have shown that AMPKα activation also confers beneficial effects on inflammation and oxidative stress.^[^ ^10^, ^11^, ^12^ ^]^ Hu et al. previously demonstrated that AMPKα activation significantly suppressed doxorubicin‐induced oxidative damage in the myocardium.^[^ ^13^ ^]^ In addition, activating AMPKα significantly inhibited inflammatory response and the activation of inflammasome in aging mice.^[^ ^14^ ^]^ Consistent with the protective role of AMPKα against inflammation and oxidative damage, Hou et al. determined that AMPKα activation prevented renal inflammation and mitochondrial ROS production, ultimately improving DN.^[^ ^15^ ^]^ And Fan et al. showed that activating AMPKα alleviated high glucose‐induced oxidative stress, thereby preventing podocyte apoptosis.^[^ ^16^ ^]^ These findings identify AMPKα as an attractive therapeutic target to treat DN.

Golgi membrane protein 1 (GOLM1, also known as GP73), a Golgi type II transmembrane protein, is mainly expressed at the Golgi apparatus and cycles among membranous compartments, which is then released to the extracellular space after being cleaved by the convertase furin.^[^ ^17^ ^]^ GOLM1 plays critical roles in tumorigenesis and is strongly associated with poor patient prognosis. Results from Qin's laboratory revealed that GOLM1 was the leading upregulated gene in hepatocellular carcinoma (HCC) with extrahepatic metastasis, and that GOLM1 overexpression promoted HCC growth and metastasis.^[^ ^18^, ^19^ ^]^ Recent studies indicated that GOLM1 is related with inflammation and oxidative stress. Pu et al. found that *Golm1*‐deficient mice are susceptible to mucosal inflammation.^[^ ^20^ ^]^ In addition, serum GOLM1 correlated with hepatic inflammation, and participated in the progression of hepatic Wilson disease.^[^ ^21^, ^22^ ^]^ Lin et al. recently demonstrated that the differentially expressed genes by GOLM1 deficiency are mainly enriched to biological processes of oxidative stress and angiogenesis.^[^ ^23^ ^]^ Based on these findings, we herein try to investigate the role and mechanism of GOLM1 in DN using gain‐ and loss‐of‐function approaches.

## 2 Experimental Section

### 2.1 Animals and Experimental Design

Male C57BL/6 mice aged 8–10 weeks were purchased from Beijing HFK Bioscience Co., Ltd, and fed with water and food with free access. To induce type 1 diabetes mellitus (T1DM)‐related DN, mice were intraperitoneally injected with 50 mg kg^−1^ streptozotocin (STZ) dissolved in 100 mmol L^−1^ sodium citrate buffer for 5 consecutive days, and then sacrificed 20 weeks post‐STZ injection.^[^ ^5^ ^]^ To induce T2DM‐related DN, mice were fed with high fat diet (HFD, 60% kcal from fat) for 24 weeks.^[^ ^5^ ^]^ In addition, mice were also fed with an HFD diet for 4 weeks, and then intraperitoneally injected with STZ (50 mg kg^−1^) daily for 3 days, followed by continued HFD feeding for an additional 12 weeks to induce T2DM‐induced DN by STZ/HFD.^[^ ^24^ ^]^ For the generation of podocyte‐specific GOLM1 knockout (*Golm1* ^pKO^) mice, GOLM1‐floxed (*Golm1* ^fl/fl^) mice were constructed and bred with C57BL/6J background podocyte‐specific podocin promoter (Podocin‐cre, Stock No. 0 08205, Jackson Laboratory). *Golm1* ^fl/fl^ mice genotyping was performed using following primers: 5′arm forward, 5′‐TAGGCAGCACTCCTCTGTGGTTTCT‐3′, 5′arm reverse, 5′‐AGGCTGGGCAGATCAAAGACAA‐3′; 3′arm forward, 5′‐GCTGAGGGTGGGACACTTGTGTAGT‐3′, 3′arm reverse, 5′‐CAATCTCAGGTCACCGGGTTTAGC‐3′. To generate podocyte‐specific GOLM1 overexpression (*Golm1* ^pTG^) mice, murine *Golm1* cDNA was synthesized and cloned under podocyte promoter, which was then linearized and microinjected into fertilized mouse embryos. GOLM1 is defined as a GTPase‐activating protein (GAP) contributing to the pathogenesis of nonobese nonalcoholic fatty liver disease, and *Golm1* ^pKO^ mice were overexpressed with GOLM1 WT or a GAP‐inactive GOLM1 RQ to investigate whether the role of GOLM1 on DN was dependent on its GAP activity.^[^ ^25^ ^]^ For AMPKα inhibition, STZ‐ or HFD‐treated mice were intraperitoneally injected with 20 mg kg^−1^ dorsomorphin (DMP, #S7840, Selleck, Houston, TX) every other day for 2 weeks before the study terminated.^[^ ^26^ ^]^ To silence peroxisome proliferator activated receptor γ (PPARγ, encoded by *Pparg* gene) in mice, *Golm1* ^pKO^ mice were injected with adeno‐associated virus serotype 9 (AAV9)‐packaged PPARγ knockdown plasmid (sh*Pparg*, #MSH094324, GeneCopoeia, Rockville, MD) under a podocin promoter with a concentration of 1 × 10^11^ vg mL^−1^ through the renal vein as previously described.^[^ ^27^ ^]^ Briefly, mice were anesthetized with the left kidney exposed. Next, renal vein was clamped, and then injected with AAV9 vectors using a 31G needle. After 15 min, the clamp was removed and the incision was sutured. To neutralize GOLM1 in vivo, mice were intravenously injected with anti‐GOLM1 (1 mg kg^−1^) every other day at the last month before the study terminated.^[^ ^28^ ^]^ All animal experiments were approved by the Institutional Animal Care and Use Committee of the hospital.

### 2.2 Morphological Studies

Periodic‐acid‐schiff (PAS) staining was performed on paraffin‐embedded sections according to standard protocols as previously described, and glomerular surface area as well as mesangial area were determined using the Image‐Pro Plus 6.0 software (Media Cybernetics, Rockville, MD).^[^ ^7^ ^]^ To determine renal fibrosis, Masson's trichrome staining was performed, and fibrotic area was quantified by the Image‐Pro Plus 6.0 software.

### 2.3 Immunofluorescence Staining, Dihydroethidium (DHE) Staining and TdT‐Mediated dUTP Nick‐End Labeling (TUNEL) Staining

For immunofluorescence staining, paraffin‐embedded sections were dewaxed, hydrated, and then subjected to antigen repair. Next, the sections were incubated with antitumor necrosis factor‐α (TNF‐α) at a dilution of 1:100 at 4 °C overnight, and then stained with Alexa Fluor 488‐conjugated secondary antibody at 37 °C for 1 h.^[^ ^29^ ^]^ Nucleus were counterstained with DAPI, and then, the images were captured under fluorescence microscope in a blind manner. To evaluate the generation of free radicals, fresh frozen renal sections were prepared and subjected to DHE staining as previously described.^[^ ^8^, ^30^ ^]^ Briefly, fresh frozen sections were incubated with 2 µmol L^−1^ DHE at 37 °C for 30 min, and then, the images were captured under fluorescence microscope in a blind manner. Fresh frozen renal sections were also subjected to TUNEL staining to label apoptotic nuclei according to standard protocols.^[^ ^31^ ^]^

### 2.4 Serum and Urinary Assays

Creatinine (Cr), blood urea nitrogen (BUN), total cholesterol (TC), and triglyceride (TG) levels were determined using Creatinine Assay Kit (#C011‐2‐1, Nanjing Jiancheng Bioengineering Institute, Jiangsu, China), Urea Assay Kit (#C013‐2‐1), Total Cholesterol Assay Kit (#A111‐1‐1) or Triglyceride Assay Kit (#A110‐1‐1, Nanjing) according to the manufacturers' instructions. Urinal albumin was detected using Albumin Assay Kit (#A028‐2‐1), and then, urine albumin‐to‐creatinine ratio (UACR) was calculated. Fasting blood glucose (FBG) level was assayed with a One Touch Ultra Easy glucometer (Life Scan, Wayne, PA). Mouse (#MBS453157, MyBioSource, San Diego, CA) and human (#MBS2020138, MyBioSource) GOLM1 ELISA Kits were used to detect serum GOLM1 level in mice and human according to the manufacturers' instructions.

### 2.5 Measurement of Blood Pressure

Systolic blood pressure (SBP) and diastolic blood pressure (DBP) were measured using the Softron BP‐2010 tail‐cuff system (Softron, Tokyo, Japan) as previously described.^[^ ^32^ ^]^ Mice were trained for 5 consecutive days, and then subjected to blood pressure measurement.

### 2.6 Biochemical Analysis

To evaluate renal fibrosis, the level of renal hydroxyproline was determined using the Hydroxyproline Assay Kit (#A030‐3‐1, Nanjing Jiancheng Bioengineering Institute) according to the manufacturers' instructions. Briefly, fresh kidneys were homogenized in 6 mol L^−1^ HCL, and then hydrolyzed at 95 °C for 5 h. Next, pH value of the buffer was modulated to 6.0–6.8, and incubated with indicating reagents at 60 °C for 15 min. Subsequently, the lysates were centrifuged at 3500 rpm for 10 min, and the supernatant was collected and measured at 550 nm. Renal hydrogen peroxide (H_2_O_2_) and superoxide anion (O_2_^–^) levels were determined using Intracellular Hydrogen Peroxide Assay (#MAK164, Sigma‐Aldrich) and Superoxide Anion Assay Kit (#CS1000, Sigma‐Aldrich) according to the manufacturers' instructions. To measure malondialdehyde (MDA), 3‐Nitrotyrosine (3‐NT), and 8‐hydroxy 2 deoxyguanosine (8‐OHdG), commercial kits (#ab118970, #ab116691, #ab201734, Abcam, Cambridge, UK) were used. To measure NF‐κB activity, nuclear extracts were prepared and mixed with a biotinylated oligo containing the NF‐κB transcription factor‐binding site, which was then transferred to a 96‐well streptavidin coated plate to capture the biotinylated oligo (#40 098, Active Motif, Carlsbad, CA). Next, the primary antibody of the transcription factor is added, followed by an incubation with HRP‐secondary antibody and Developing Solution, respectively. Finally, the absorbance was measured at 450 nm with a reference wavelength of 655 nm using a spectrophotometer. To measure TNF‐α, interleukin‐1β (IL‐1β) and IL‐6 levels, commercial kits (#ab208348, #ab197742, #ab222503, Abcam) were used. Caspase‐3 activity was measured using the Caspase‐3 Cellular Activity Assay Kit (#235 419, Sigma‐Aldrich) according to the manufacturers' instructions. The activity of protein kinase A (PKA) was evaluated by a PKA Kinase Activity Assay Kit (#ab139435, Abcam). Serum levels of alanine aminotransferase (ALT), aspartate aminotransferase (AST), troponin T, creatine kinase‐MB (CK‐MB), and creatine kinase (CK) were detected using the following commercial kits according to the manufacturers' instructions: Mouse ALT ELISA Kit (#ab282882, Abcam), Mouse AST ELISA Kit (#ab263882, Abcam), Mouse Troponin T ELISA Kit (#CSB‐EL024016MO, CUSABIO, Houston, TX), Mouse Creatine Kinase MB ELISA Kit (#ab285231, Abcam), and Creatine Kinase Activity Assay Kit (#ab155901, Abcam).

### 2.7 Cell Isolation and Treatment

Primary podocytes were isolated as previously described, and the identify and purity of primary podocytes were verified by the immunostaining of podocin, a biomarker of podocytes.^[^ ^33^ ^]^ Briefly, fresh kidneys were minced and incubated with 1 mg mL^−1^ collagenase A and DNAse I for 1 h, which was then strained through a 100 µm strainer. Next, the cells were resuspended in 45% Percoll solution and centrifuged at 4 °C for 1 h. After removing the Percoll solution, cell pellet was resuspended and propagated on type I collagen‐coated plates. To imitate diabetes in vitro, podocytes were stimulated with high glucose (HG, 25 mmol L^−1^) for 72 h or normal glucose (NG, 5 mmol L^−1^) plus mannitol (25 mmol L^−1^) as the control. To investigate whether the extracellular fragment is the active form of GOLM1, *Golm1* ^pKO^ podocytes were treated with recombinant GOLM1 (rGOLM1, 32 nmol L^−1^) or vehicle for 4 h under HG stimuli.^[^ ^28^ ^]^ To neutralize extracellular GOLM1, podocytes were incubated with anti‐GOLM1 (1 µg mL^−1^) for 24 h.^[^ ^28^ ^]^ In addition, podocytes were also transfected with small interfering RNA against FURIN (si*Furin*, 50 nmol L^−1^) or epidermal growth factor receptor (EGFR) to knock down FURIN or EGFR for 4 h, and then cultured for 24 h before HG stimuli. To overexpress GOLM1 WT, GOLM1 RVAA, or GOLM1 RQ, podocytes were infected with adenovirus for 4 h, and then cultured for 24 h before HG stimuli. To inhibit AMPKα in vitro, podocytes were incubated with 20 µmol L^−1^ DMP for 72 h with or without HG stimulation.^[^ ^26^ ^]^ For PPARγ inhibition, cells were incubated with 10 µmol L^−1^ GW9662 (#S2915, Selleck) for 72 h.^[^ ^12^ ^]^ In addition, rat glomerular endothelial cells, rat mesangial cells, RAW264.7 cells and rat proximal tubule epithelial cells were cultured as previously described, and stimulated with HG to imitate hyperglycemic stimuli in vitro.^[^ ^24^ ^]^ Moreover, podocytes and nonpodocytes (endothelial cells, mesangial cells, and immune cells, etc.,) were also isolated as described previously.^[^ ^34^, ^35^ ^]^ Briefly, fresh kidneys were minced and digested in 0.2 mg mL^−1^ Liberase TL, 100 U mL^−1^ DNAse I in RPMI 1640 medium by shaking at 37 °C for 30 min, which was then passed through an 18G needle for 10 times to further dissociate the tissue. The cell suspensions were passed through a 100 µm, then a 40 µm cell strainer to remove cell debris after the inactivation of enzymes, and was then pelleted by centrifugation at 4 °C for 5 min. Next, cells were resuspended and incubated with two rabbit anti‐Nephrin antibodies at 4 °C for 1 h, and then reacted with antirabbit microbeads as well as Alexa Fluora 594‐conjugated AffiniPure Donkey Anti‐Rabbit IgG antibodies at 4 °C for 30 min. Cells were pelleted, washed and applied to MACS LS columns to separate microbead‐bound podocytes from the other kidney cells, while cells not retained by the magnetic field were collected as nonpodocytes fractions.

### 2.8 Cell Viability and Cell Damage

To determine cell viability, cells were incubated with 10 µL cell counting kit‐8 (CCK‐8) solution (#C0037, Beyotime, Shanghai, China) per well at 37 °C for 1 h, and then, the absorbance was measured at 450 nm with a reference wavelength of 650 nm.^[^ ^36^, ^37^, ^38^ ^]^ To evaluate cell damage in vitro, cells were incubated with the reaction mix and measured at 450 nm using a Lactate Dehydrogenase (LDH) Assay Kit (#ab102526, Abcam).^[^ ^39^ ^]^

### 2.9 Analysis of ROS Level in Cells

To measure ROS generation in cells, podocytes were incubated with 10 µmol L^−1^ DCFH‐DA at 37 °C for 20 min using the Reactive Oxygen Species Assay Kit (#S0033, Beyotime), and then, the intensity was recorded at 488/525 nm as previously described.^[^ ^13^, ^40^ ^]^

### 2.10 Western Blot and Immunoprecipitation (IP)

Total proteins were extracted using RIPA buffer, and then, cell lysates were centrifuged at 12 000 g for 10 min.^[^ ^41^, ^42^ ^]^ Protein concentrations were determined by a bicinchoninic acid protein assay kit. Next, extracted proteins were separated by SDS‐PAGE and transferred to PVDF membranes. The membranes were blocked in 5% skim milk at room temperature for 1 h, and then, incubated with the following primary antibodies at 4 °C overnight: anti‐GOLM1 (#sc‐365817, Santa Cruz, Dallas, TX), anti‐GAPDH (#sc‐365062, Santa Cruz), anti‐p‐AMPKα (#2535, CST, Danvers, MA), anti‐t‐AMPKα (#5832, CST), anti‐PPARγ (#2443, CST). On the next day, the membranes were incubated with HRP‐conjugated secondary antibodies and visualized by an ECL reagent. Densitometric analysis of western blot images was conducted by Image Lab software (version 6.0). For IP assay, podocytes lysates were immunoprecipitated with anti‐GOLM1 or IgG, and then were used for the detection of EGFR as previously described.^[^ ^42^ ^]^

### 2.11 Quantitative Real‐Time PCR

For quantitative real‐time PCR analysis, total RNA was extracted using TRIzol (Invitrogen), and cDNA was prepared by a high‐capacity cDNA synthesis Kit (#4 368 814, Applied Biosystems, Foster City, CA). Next, quantitative real‐time PCR analysis was performed on a Bio‐Rad CFX96 Touch Real‐Time PCR Detection System using the SYBR Green reagent.^[^ ^43^, ^44^, ^45^ ^]^ The primer sequences were as following: *Golm1* forward, 5′‐CGTCGCAGCATGAAGTCTC‐3′, reverse, 5′‐CAGTAGTTGAAGCCTAGCACAAT‐3′; *Bax* forward, 5′‐TGAAGACAGGGGCCTTTTTG‐3′, reverse, 5′‐AATTCGCCGGAGACACTCG‐3′; *Bcl‐2* forward, 5′‐ATGCCTTTGTGGAACTATATGGC‐3′, reverse, 5′‐GGTATGCACCCAGAGTGATGC‐3′; *Ppara* forward, 5′‐AGAGCCCCATCTGTCCTCTC‐3′, reverse, 5′‐ACTGGTAGTCTGCAAAACCAAA‐3′; *Ppard* forward, 5′‐TCCATCGTCAACAAAGACGGG‐3′, reverse, 5′‐ACTTGGGCTCAATGATGTCAC‐3′; *Pparg* forward, 5′‐TCGCTGATGCACTGCCTATG‐3′, reverse, 5′‐GAGAGGTCCACAGAGCTGATT‐3′.

### 2.12 RNA‐Sequencing

Total RNA was extracted using an RNeasy Mini Kit and quantified by the Nanodrop 2000c. After read quality control, mapping and normalization steps on the raw sequencing reads, the data were compared to identify differentially expressed genes (DEGs) with an adjust *P* value less than 0.05 and fold change > 2 or < ‐2. Next, the DEGs were integrated to enrichment analysis for canonical pathway and gene ontology.

### 2.13 Human Renal Samples

Human renal biopsy samples were obtained from the hospital, and the experimental protocols were approved by the Institutional Review Board of the Ethics Committee (Approval No. ZSLL‐KY‐2024‐015‐01). Written informed consent was obtained from each patient and the lineal consanguinity. These patients were diagnosed by clinical characterizes and pathological examinations. Control samples were obtained from the healthy kidney poles of individuals without diabetes or kidney diseases who underwent tumor nephrectomies. Baseline characteristics of DN and control participants were as following (**Table** **1**).

**Table 1:** Baseline characteristics of DN and control participants.

	Control	DN
Gender [male/female]	17/14	15/16
Age [years]	62.17 ± 8.11	59.46 ± 5.82
FBG [mmol L^−1^]	4.34 ± 0.62	9.77 ± 1.15
HBA1c [%]	4.91 ± 0.59	7.44 ± 0.65
TC [mmol L^−1^]	4.01 ± 0.35	5.11 ± 0.62
TG [mmol L^−1^]	2.11 ± 0.19	3.64 ± 0.38
Cr [µmol L^−1^]	82.75 ± 10.74	271.42 ± 44.03
eGFR [mL min^−1^ 1.73 m^−2^]	109.04 ± 13.11	28.05 ± 4.22
DBP [mmHg]	78.64 ± 9.04	92.77 ± 10.56
SBP [mmHg]	150.31 ± 17.24	114.62 ± 12.89

### 2.14 Statistical Analysis

All data are represented as mean ± SD, and analyzed using SPSS software (version 21.0). Differences between two independent groups were evaluated two‐tailed Student's *t*‐test, and significant differences among three or more groups were determined by one‐way ANOVA or two‐way ANOVA followed with Tukey's post hoc test. Correlations were evaluated by Spearman's rank correlation test. A *P* value of less than 0.05 was considered significant.

## 3 Results

### 3.1 GOLM1 Expression is Elevated During DN Progression and Correlates with Renal Dysfunction

**Figure 1:**
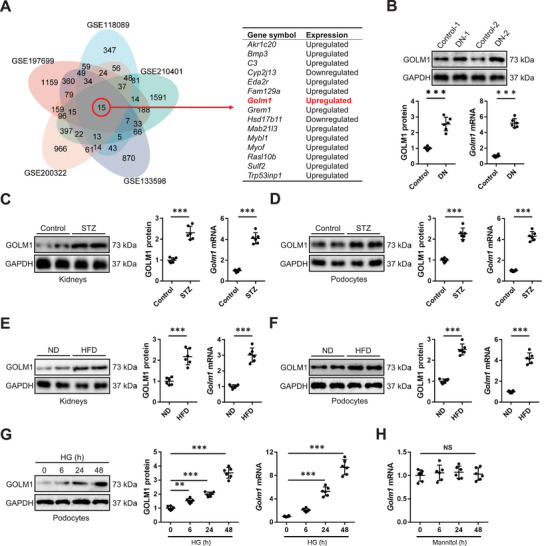
GOLM1 expression is elevated during DN progression. A) Overlapped DEGs in five GEO datasets. B) GOLM1 mRNA and protein levels in kidneys from DN patients or control participants. C,D) GOLM1 mRNA and protein levels in kidneys or podocytes from STZ‐treated mice. E,F) GOLM1 mRNA and protein levels in kidneys or podocytes from HFD‐treated mice. G,H) GOLM1 mRNA and protein levels in podocytes treated with HG or mannitol. *N* = 6 per group. *P < 0.05, **P < 0.01, ***P < 0.001 versus the matched groups. NS indicates no significance.

**Figure 2:**
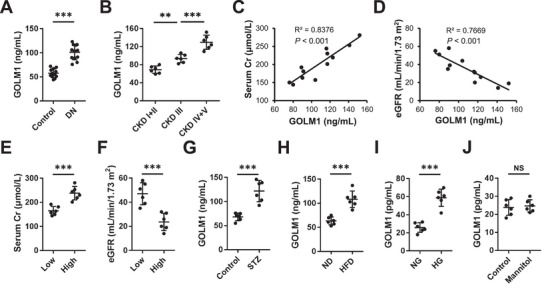
GOLM1 expression positively correlates with renal dysfunction. A) Circulating GOLM1 level in DN patients or control participants (*N* = 12). B) Circulating GOLM1 level in DN patients with different CKD stage. C,D) Correlations between circulating GOLM1 level and serum Cr as well as eGFR (*N* = 12). E,F) Serum Cr and eGFR levels in DN patients with different circulating GOLM1 concentration. G,H) Circulating GOLM1 level in STZ‐ and HFD‐treated mice or matched control mice. I,J) GOLM1 level in the medium from podocytes treated with HG or mannitol. *N* = 6 per group. *P < 0.05, **P < 0.01, ***P < 0.001 versus the matched groups. NS indicates no significance.

To determine the key molecules in DN, we integrated five GEO datasets and identified 15 overlapped DEGs in DN mice (**Figure** **1**A).^[^ ^28^ ^]^ The roles of BMP3, C3, EAD2R, FAM129A (also known as NIBAN), GREM1, MYBL1, and SULF2 in diabetic nephrology or other chronic kidney diseases have been identified in previous studies. Akr1c20, Cyp2j13, and Trp53inp1 were not expressed in human genomes. MYOF, RASL10B, and MAB21L3 were expressed in kidneys at low levels, and more importantly, no previous evidences have been obtained to indicate their potential involvements in diabetic nephrology. HSD17B11 was abundantly expressed in the kidney, and actually, its role in diabetic nephrology is investigating in another study from our research group. GOLM1 is defined as a novel glucogenic hormone and contributes to hyperglycemia in patients, so we selected GOLM1 for study. Consistent with the sequencing data, we found that GOLM1 protein level was elevated in kidneys from DN patients (Figure 1B). Next, we used STZ to construct T1DM‐related DN in mice. As shown in Figure 1C, STZ injection resulted in increased GOLM1 mRNA and protein levels in diabetic kidneys. Podocytes play critical roles in DN progression, and our data showed that GOLM1 was abundantly expressed in podocytes under basal conditions (Figure S1A,B, Supporting Information). Then, we isolated primary podocytes from STZ‐treated or control mice to investigate whether podocyte GOLM1 expression was altered during DN. As shown in Figure 1D, we found that GOLM1 mRNA and protein levels were significantly elevated in podocytes from STZ‐injected mice. In addition, we also fed mice with HFD to construct T2DM‐related DN. Consistently, GOLM1 expression was also upregulated in diabetic kidneys and podocytes from HFD‐treated mice (Figure 1E,F). To clarify the effect of hyperglycemia on GOLM1 expression in podocytes in vitro, we treated primary podocytes with HG. As shown in Figure 1G,H, GOLM1 mRNA and protein levels were significantly increased in HG‐stimulated podocytes, instead of mannitol‐treated podocytes. However, the expression of GOLM1 was unaltered by hyperglycemic stimuli in other kidney cells (Figure S1C–F, Supporting Information). Consistently, GOLM1 mRNA levels were dramatically elevated in podocytes from diabetic kidneys, rather than nonpodocytes (Figure S1G,H, Supporting Information). Previous studies have shown that GOLM1 is a novel hormone; therefore, we investigated whether circulating GOLM1 could be a biomarker of DN. As shown in **Figure** **2**A,B, serum GOLM1 level was elevated in DN patients, and also positively correlated with chronic kidney disease (CKD) stage. Meanwhile, we found that circulating GOLM1 concentration positively correlated with serum Cr level, but negative correlated with eGFR in diabetic patients (Figure 2C,D). Next, we divided DN patients using the median of circulating GOLM1 level. As shown in Figure 2E,F, DN patients with higher GOLM1 concentration exhibited higher serum Cr and lower eGFR. Consistent with the clinical data, we also found that serum GOLM1 level was elevated in diabetic mice with STZ or HFD treatment (Figure 2G,H). Moreover, stimulation with HG, instead of mannitol, significantly facilitated GOLM1 secretion from podocyte to the medium (Figure 2I,J). Collectively, our results reveal that GOLM1 expression is elevated during DN progression and correlates with renal dysfunction.

### 3.2 Podocyte‐Specific GOLM1 Ablation Prevents T1DM‐ and T2DM‐Induced DN in Mice

**Figure 3:**
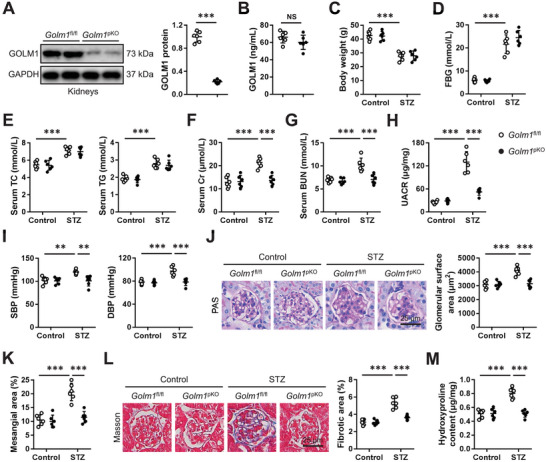
Podocyte‐specific GOLM1 ablation prevents DN in STZ‐treated mice. A) GOLM1 protein levels in kidneys from *Golm1* ^pKO^ or *Golm1* ^fl/fl^ mice. B) Circulating GOLM1 levels in *Golm1* ^pKO^ or *Golm1* ^fl/fl^ mice. C) Body weight among groups. D,E) The levels of FBG, TC, and TG among groups. F,G) The levels of serum Cr and BUN. H) Quantification of UACR. I) Blood pressure among groups. J,K) Representative PAS images, and quantification of glomerular surface area and mesangial area. L) Representative Masson's trichrome staining images and quantification of fibrotic area. M) Quantification of renal hydroxyproline content. *N* = 6 per group. *P < 0.05, **P < 0.01, ***P < 0.001 versus the matched groups. NS indicates no significance.

To clarify the role of GOLM1 during DN progression, we established podocyte‐specific GOLM1 ablated mice (**Figure** **3**A). Interestingly, podocyte‐specific ablation of GOLM1 did not affect circulating GOLM1 level in mice, indicating a local role of GOLM1 within kidneys (Figure 3B). Accordingly, body weight and systemic metabolic parameters, including body weight, FBG, TC, and TG, were also unaffected by podocyte‐specific GOLM1 deficiency (Figure 3C–E). Yet, diabetes‐induced renal dysfunction was significantly ameliorated in *Golm1* ^pKO^ mice, as determined by decreased serum Cr, BUN, and UCAR levels (Figure 3F–H). Consistent with the improvement of renal function, DN‐related increases of DBP and SBP were also decreased by podocyte‐specific GOLM1 ablation (Figure 3I). Histologic analysis of kidneys implied that podocyte‐specific deletion of GOLM1 significantly attenuated glomerulomegaly and mesangial region expansion in diabetic mice (Figure 3J,K). Meanwhile, *Golm1* ^pKO^ mice showed lower renal fibrosis upon STZ injection (Figure 3L,M). To further confirm the role of podocyte GOLM1 in DN, we also constructed T2DM‐induced DN through feeding mice with HFD for 24 weeks. As shown in Figure S2A–C (Supporting Information), HFD‐induced increases of serum Cr, BUN, and UACR were significantly reduced in *Golm1* ^pKO^ mice. Podocyte‐specific GOLM1 ablation also prevented glomerulomegaly, mesangial region expansion and renal fibrosis in HFD‐fed mice (Figure S2D–G, Supporting Information). Collectively, our results reveal that podocyte‐specific GOLM1 ablation prevents T1DM‐ and T2DM‐induced DN in mice.

### 3.3 Podocyte‐Specific GOLM1 Overexpression Facilitates T1DM‐ and T2DM‐Induced DN in Mice

**Figure 4:**
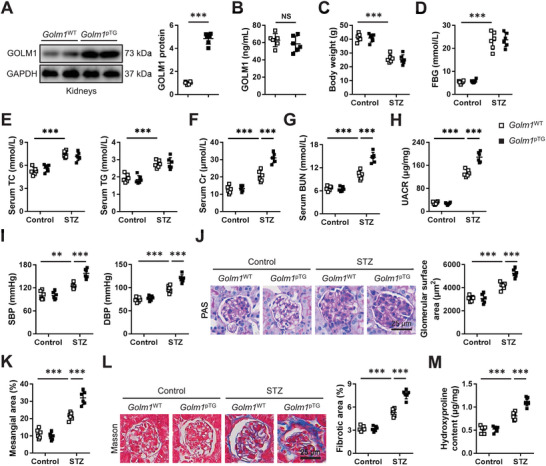
Podocyte‐specific GOLM1 overexpression facilitates DN in STZ‐treated mice. A) GOLM1 protein levels in kidneys from *Golm1* ^pTG^ or *Golm1* ^WT^ mice. B) Circulating GOLM1 levels in *Golm1* ^pTG^ or *Golm1* ^WT^ mice. C) Body weight among groups. D,E) The levels of FBG, TC, and TG among groups. F,G) The levels of serum Cr and BUN. H) Quantification of UACR. I) Blood pressure among groups. J,K) Representative PAS images, and quantification of glomerular surface area and mesangial area. L) Representative Masson's trichrome staining images and quantification of fibrotic area. M) Quantification of renal hydroxyproline content. *N* = 6 per group. *P < 0.05, **P < 0.01, ***P < 0.001 versus the matched groups. NS indicates no significance.

Next, we established podocyte‐specific GOLM1 overexpressed mice to further validate the role of GOLM1 during DN progression (**Figure** **4**A,B). As shown in Figure 4C–E, body weight and systemic metabolism were unaffected in *Golm1* ^pTG^ mice upon STZ injection. Yet, the increased serum Cr, BUN, and UACR levels in diabetic mice were further aggravated by podocyte‐specific GOLM1 overexpression (Figure 4F–H). Meanwhile, diabetes‐related hypertension was exacerbated in *Golm1* ^pTG^ mice (Figure 4I). PAS staining results suggested that podocyte‐specific GOLM1 overexpression further facilitated glomerulomegaly and mesangial region expansion in diabetic mice (Figure 4J,K). Moreover, diabetes‐related renal fibrosis was also exacerbated in *Golm1* ^pTG^ mice, as determined by Masson's trichrome staining and quantification of hydroxyproline level (Figure 4L,M). Meanwhile, we also evaluated the role of GOLM1 in HFD‐induced DN in mice. As shown in Figure S3A–C (Supporting Information), the increases of serum Cr, BUN, and UACR in HFD‐treated mice were further amplified by podocyte‐specific GOLM1 overexpression. HFD‐induced glomerulomegaly, mesangial region expansion and renal fibrosis were also exacerbated in *Golm1* ^pTG^ mice (Figure S3D–G, Supporting Information). Collectively, our results reveal that podocyte‐specific GOLM1 overexpression facilitates T1DM‐ and T2DM‐induced DN in mice.

### 3.4 Podocyte GOLM1 Inhibits Inflammation and Oxidative Damage in DN Mice

**Figure 5:**
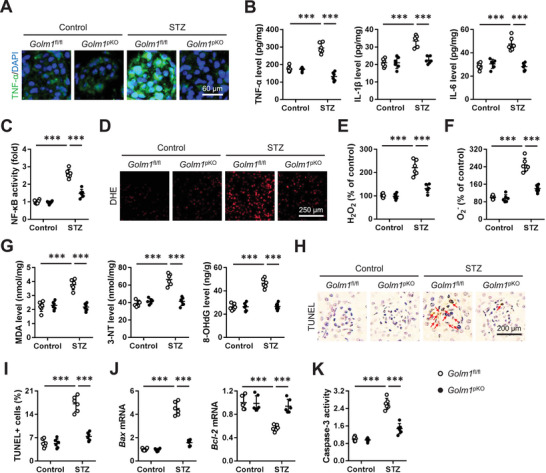
Podocyte‐specific GOLM1 ablation inhibits inflammation and oxidative damage in STZ‐treated mice. A) Representative TNF‐α immunofluorescence staining images. B) The levels of renal TNF‐α, IL‐1β, and IL‐6. C) Relative NF‐κB transcription activity in kidneys. D) Representative DHE staining images. E,F) The levels of H_2_O_2_ and O_2_^‐^ in kidneys. G) The levels of renal MDA, 3‐NT and 8‐OHdG. H,I) Representative TUNEL staining images and quantification of TUNEL+ cells. J) Relative *Bax* and *Bcl‐2* mRNA levels in kidneys. K) Relative caspase‐3 activity in kidneys. *N* = 6 per group. *P < 0.05, **P < 0.01, ***P < 0.001 versus the matched groups.

Inflammation is a key initiator of DN, and inhibiting inflammation serves as an attractive approach to block DN progression. Immunofluorescence staining results indicated that the increased TNF‐α expression was significantly reduced in *Golm1* ^pKO^ mice upon STZ injection (**Figure** **5**A). Meanwhile, STZ‐induced elevations of TNF‐α, IL‐1β, and IL‐6 were also inhibited by podocyte‐specific GOLM1 ablation (Figure 5B). NF‐κB is a central transcription factor of inflammation that plays critical roles in DN progression. We found that podocyte‐specific GOLM1 ablation significantly suppressed NF‐κB transcription activity in diabetic mice (Figure 5C). Oxidative damage is the other feature and contributor of DN. As shown in Figure 5D–F, SZT injection resulted in significant increases of free radicals in kidneys, which were prevented in *Golm1* ^pKO^ mice. Accordingly, podocyte‐specific GOLM1 ablation significantly reduced oxidative damage in diabetic kidneys, as evidenced by the decreased MDA, 3‐NT and 8‐OHdG levels (Figure 5G). Inflammation and oxidative damage synergistically facilitated cell apoptosis and renal dysfunction. As shown in Figure 5H–K, STZ‐induced increase of cell apoptosis was reduced in *Golm1* ^pKO^ mice, as evidenced by decreased TUNEL+ cells, *Bax* mRNA, caspase‐3 activity, and increased *Bcl‐2* mRNA. In contrast with the loss‐of‐function study, we found that the increased TNF‐α, IL‐1β, and IL‐6 levels in diabetic kidneys were further augmented by podocyte‐specific GOLM1 overexpression (Figure S4A, Supporting Information). STZ‐induced oxidative damage was also exacerbated in *Golm1* ^pKO^ mice, as evidenced by increased H_2_O_2_, O_2_^‐^, MDA, 3‐NT, and 8‐OHdG levels (Figure S4B–D, Supporting Information). Accordingly, podocyte‐specific GOLM1 overexpression also facilitated cells apoptosis in STZ‐injected mice (Figure S4E–G, Supporting Information). We also validated the role of podocyte GOLM1 in HFD‐induced DN in mice. As shown in Figure S5A–C (Supporting Information), podocyte‐specific GOLM1 ablation significantly blocked HFD‐induced apoptosis, inflammation, and oxidative damage in kidneys. While HFD‐induced apoptosis, inflammation, and oxidative damage in kidneys were further augmented in *Golm1* ^pTG^ mice (Figure S5E,F, Supporting Information). In addition, we also used a combination of HFD and STZ to imitate DN in vivo. The data showed that podocyte‐specific GOLM1 ablation prevented, whereas podocyte‐specific GOLM1 overexpression facilitated inflammation, oxidative stress, and renal dysfunction in STZ/HFD‐treated mice (Figure S6A–H, Supporting Information). Collectively, our results reveal that podocyte GOLM1 inhibits inflammation and oxidative damage in DN mice.

### 3.5 Podocyte GOLM1 Aggravates High Glucose‐Induced Inflammation and Oxidative Damage in an Autocrine‐Dependent Manner In Vitro

Subsequently, we explored the role of GOLM1 in high glucose‐induced inflammation and oxidative damage in primary podocytes in vitro. As shown in Figure S7A,B (Supporting Information), HG stimulation resulted in significant cell loss and damage, which were inhibited in *Golm1* ^pKO^ podocytes, as evidenced by the increased cell viability and decreased LDH releases. Meanwhile, HG‐stimulated inflammation and oxidative damage were also attenuated by GOLM1 deficiency (Figure S7C–E, Supporting Information). In contrast with the loss‐of‐function study, we found that GOLM1 overexpression further facilitated cell loss and damage upon HG stimulation (Figure S7F,G, Supporting Information). Accordingly, the increased TNF‐α, IL‐1β, IL‐6, ROS, MDA, 3‐NT, and 8‐OHdG levels in HG‐stimulated podocytes were also augmented by GOLM1 overexpression (Figure S7H–J, Supporting Information). GOLM1 is identified as a novel hormone, and we investigated whether extracellular GOLM1 was sufficient to aggravate DN in vitro.^[^ ^28^ ^]^ As shown in Figure S8A,B (Supporting Information), the incubation of *Golm1* ^pKO^ podocytes with rGOLM1 dramatically abolished the protective effects against hyperglycemic stimuli, which were blocked by the supplementation of a GOLM1 neutralizing antibody. Meanwhile, the treatment with anti‐GOLM1 also prevented HG‐induced podocyte injury in both *Golm1* ^WT^ podocytes and *Golm1* ^pTG^ podocytes (Figure S8C,D, Supporting Information). Previous studies indicated that GOLM1 was cleaved by a proprotein convertase FURIN at R^52^VRR^55^ site, and subsequently released to the extracellular space.^[^ ^46^ ^]^ Then, we knocked down the expression of FURIN to reduce the secretion of GOLM1, and the efficiency was validated by western blot (Figure S8E, Supporting Information). As expected, FURIN knockdown decreased the culture GOLM1 levels in *Golm1* ^pTG^ podocytes under HG stimuli (Figure S8F, Supporting Information). Accordingly, the increased cellular injury in *Golm1* ^pTG^ podocytes was also blocked by FURIN knockdown (Figure S8G, Supporting Information). In addition, preventing GOLM1 cleavage by mutating the R^52^VRR^55^ site to RVAA also reduced the culture GOLM1 levels in *Golm1* ^pKO^ podocytes receiving GOLM1 overexpression under HG stimuli, accompanied with a decreased cellular injury (Figure S8H–J, Supporting Information). Collectively, our results reveal that podocyte GOLM1 aggravates high glucose‐induced inflammation and oxidative damage in an autocrine‐dependent manner in vitro.

### 3.6 Podocyte‐Specific GOLM1 Ablation Attenuates T1DM‐ and T2DM‐Induced DN Through Activating AMPKα Pathway

**Figure 6:**
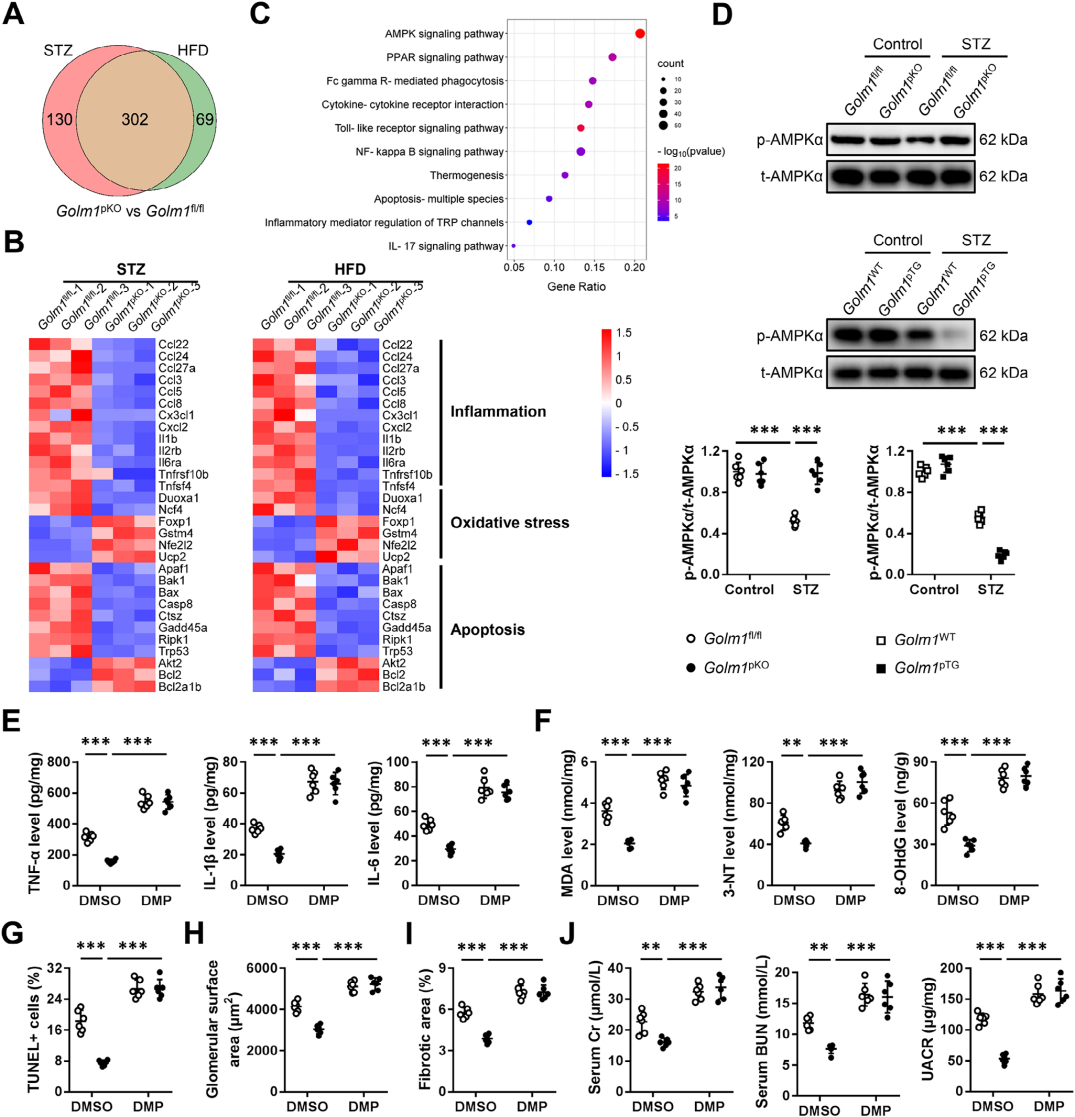
Podocyte‐specific GOLM1 ablation attenuates STZ‐induced DN through activating AMPKα pathway. A) The DEGs under STZ or HFD contexts were intersected to identify the downstream target genes of GOLM1 (*N* = 3). B) Heatmaps of inflammation‐, oxidative stress‐, and apoptosis‐related genes (*N* = 3). C) Enriched pathway analysis (*N* = 3). D) The levels of AMPKα phosphorylation in *Golm1* ^pKO^, *Golm1* ^pTG^ or matched control mice with or without STZ injection. E) The levels of renal TNF‐α, IL‐1β, and IL‐6. F) The levels of renal MDA, 3‐NT, and 8‐OHdG. G) Quantification of TUNEL+ cells. H,I) Quantification of glomerular surface area and fibrotic area. J) The levels of serum Cr, BUN, and quantification of UACR. *N* = 6 per group. *P < 0.05, **P < 0.01, ***P < 0.001 versus the matched groups.

Previous studies have identified GOLM1 as a GTPase‐activating protein (GAP) contributing to the pathogenesis of nonobese nonalcoholic fatty liver disease, and we thus determined whether the DN‐promoting role of GOLM1 depended on its GAP activity.^[^ ^25^ ^]^ To clarify the question, *Golm1* ^pKO^ podocytes or *Golm1* ^pKO^ mice were overexpressed with GOLM1 WT or a mutant GOLM1 RQ unable to catalyze GTP hydrolysis. As shown in Figure S9A–E (Supporting Information), the improved podocyte injury and renal dysfunction in *Golm1* ^pKO^ podocytes or *Golm1* ^pKO^ mice were exacerbated by the reintroduction of GOLM1 WT. Interestingly, re‐expressing GAP‐inactive GOLM1 also induced a similar phenotype as GOLM1 WT, indicating the GAP activity of GOLM1 is dispensable for its effect on DN (Figure S9A–E, Supporting Information). To investigate the potential mechanism of podocyte GOLM1 in regulating DN progression, *Golm1* ^fl/fl^ and *Golm1* ^pKO^ kidneys upon STZ or HFD treatment were collected for RNA‐sequencing analysis, and the DEGs under STZ or HFD contexts were intersected to identify the downstream target genes of GOLM1. As shown in **Figure** **6**A, 432 DEGs were identified in *Golm1* ^pKO^ versus *Golm1* ^fl/fl^ kidneys upon STZ injection, while 371 DEGs were identified in *Golm1* ^pKO^ versus *Golm1* ^fl/fl^ kidneys upon HFD treatment, and 302 overlapped DEGs were selected for further analysis. Interestingly, proinflammation, pro‐oxidant, and pro‐apoptotic genes were downregulated, while antioxidant and antiapoptotic genes were upregulated in *Golm1* ^pKO^ kidneys upon STZ or HFD treatment (Figure 6B). In addition, we found that these 302 genes were mainly enriched in AMPK signaling pathway (Figure 6C). Consistent with the sequencing data, western blot result showed that the decreased AMPKα phosphorylation in diabetic kidneys was restored by podocyte‐specific GOLM1 ablation, but further inhibited by podocyte‐specific GOLM1 overexpression (Figure 6D). To validate the involvement of AMPKα, DMP, a specific inhibitor of AMPKα was used. As shown in Figure 6E–G, AMPKα inhibition significantly abolished the anti‐inflammatory, antioxidant, and antiapoptotic effects in *Golm1* ^pKO^ kidneys upon STZ injection. Accordingly, podocyte‐specific GOLM1 ablation failed to prevent STZ‐induced glomerulomegaly and renal fibrosis in the presence of DMP (Figure 6H,I). The improved renal function in *Golm1* ^pKO^ mice upon STZ injection was also blocked by AMPKα inhibition, as evidenced by the increased serum Cr, BUN, and UACR levels (Figure 6J). Consistent with the results in STZ‐injected mice, we found that DMP treatment also abolished the anti‐inflammatory, antioxidant, and antiapoptotic effects in *Golm1* ^pKO^ kidneys upon HFD feeding (Figure S10A–C, Supporting Information). Meanwhile, the improved glomerulomegaly, renal fibrosis, and dysfunction in *Golm1* ^pKO^ mice upon HFD treatment were also blocked by AMPKα inhibition (Figure S10D–F, Supporting Information). Next, we investigated the necessity of AMPKα pathway in vitro. As shown in Figure S11A,B (Supporting Information), *Golm1* ^pKO^ podocytes were resistant to HG‐induced cell loss and damage, but failed to do so in the presence of DMP incubation. The anti‐inflammatory and antioxidant effects of *Golm1* ^pKO^ podocytes against HG stimulation were also prevented by AMPKα inhibition (Figure S11C–E, Supporting Information). Collectively, our results reveal that podocyte‐specific GOLM1 ablation attenuates T1DM‐ and T2DM‐induced DN through activating AMPKα pathway.

### 3.7 Podocyte GOLM1 Inactivates AMPKα Pathway Through Interacting with EGFR to Inhibit PPARγ

**Figure 7:**
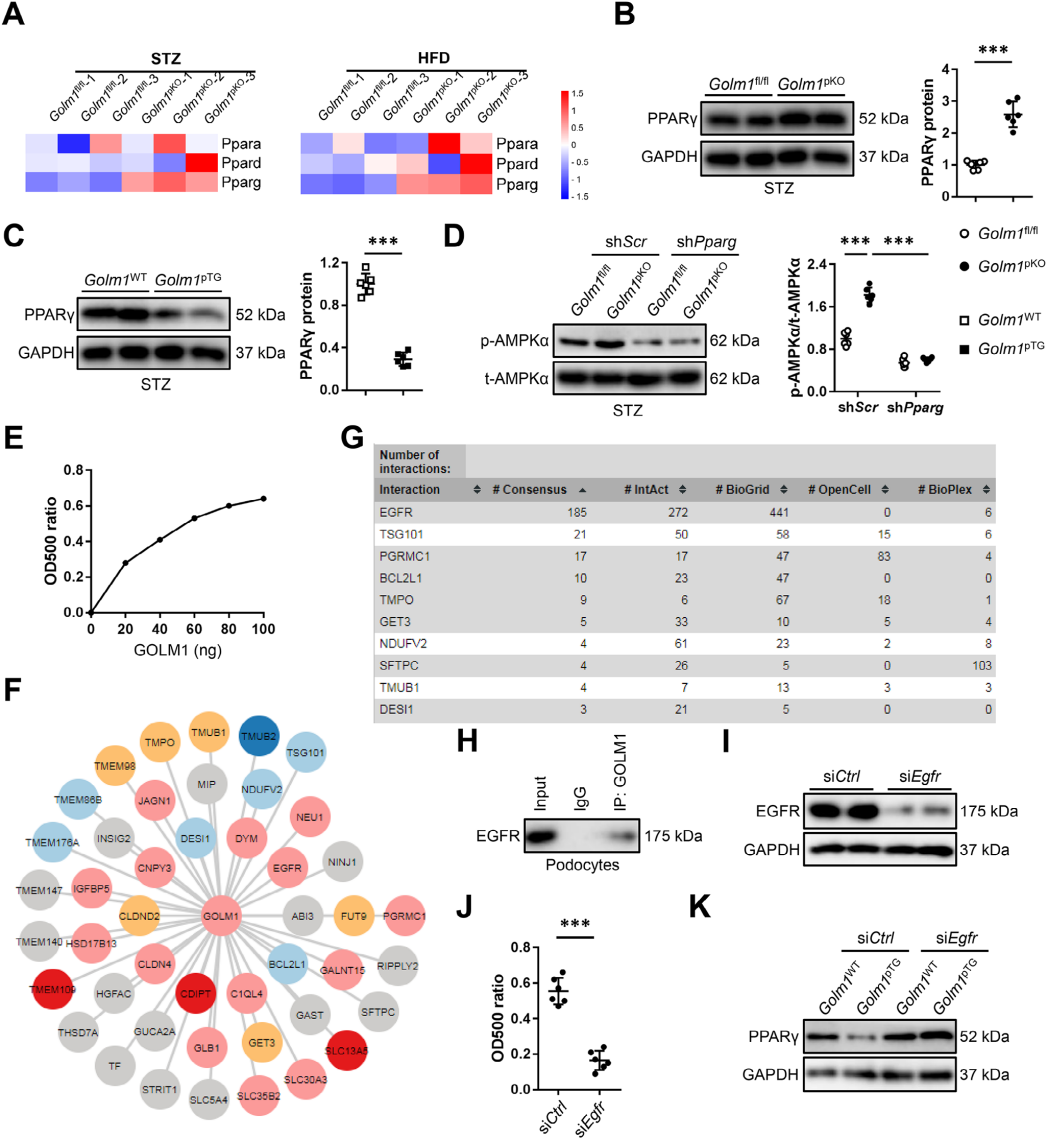
Podocyte GOLM1 inactivates AMPKα pathway through interacting with EGFR to inhibit PPARγ. A) Heatmaps of *Ppara*, *Ppard*, and *Pparg* in kidneys from *Golm1* ^pKO^ or *Golm1* ^fl/fl^ mice with STZ or HFD treatment (*N* = 3). B,C) PPARγ protein levels in kidneys from *Golm1* ^pKO^, *Golm1* ^pTG^, or matched control mice with STZ treatment. D) The levels of AMPKα phosphorylation in STZ‐treated *Golm1* ^pKO^ or *Golm1* ^fl/fl^ kidneys with or without *Pparg* silence. E) Podocytes were incubated with biotin‐labeled GOLM1, and podocytes‐interacted biotin‐GOLM1 was determined by a colorimetric assay. F) Analysis of GOLM1‐interactome from The Human Protein Atlas database. G) List of the top 10 GOLM1‐interacted proteins in the GOLM1‐interactome from The Human Protein Atlas database. H) Cell lysates from podocytes were coimmunoprecipitated with anti‐GOLM1 or IgG, and then exposed to western blot for the analysis of EGFR. I) EGFR protein levels in podocytes with or without EGFR knockdown. J) Control and EGFR‐depleted podocytes were incubated with biotin‐labeled GOLM1, and podocytes‐interacted biotin‐GOLM1 was determined by a colorimetric assay. K) GOLM1 protein levels in *Golm1* ^pTG^ podocytes with or without EGFR knockdown. *N* = 6 per group. *P < 0.05, **P < 0.01, ***P < 0.001 versus the matched groups.

We then investigated the underlying molecular basis through which podocyte GOLM1 regulated AMPKα pathway. As shown in Figure 6C, PPAR signaling pathway, a critical upstream modulator of AMPKα pathway, was also significantly altered by podocyte‐specific GOLM1 ablation. The sequencing data revealed that *Pparg*, instead of *Ppara* or *Ppard* was significantly upregulated in *Golm1* ^pKO^ kidneys upon STZ or HFD treatment (**Figure** **7**A). Consistently, results from quantitative real‐time PCR analysis indicated podocyte‐specific GOLM1 ablation elevated, while podocyte‐specific GOLM1 overexpression reduced *Pparg* mRNA levels in T1DM or T2DM mice, without affecting *Ppara* or *Ppard* expression (Figure S12A,B, Supporting Information). As shown in Figure 7B,C, PPARγ protein level was also upregulated in *Golm1* ^pKO^ kidneys, but downregulated in *Golm1* ^pTG^ kidneys upon STZ treatment. PKA is a classic activator of AMPKα pathway, and Wan et al. recently demonstrated that GOLM1 could stimulate PKA pathway.^[^ ^28^ ^]^ Therefore, we determined whether PKA was required for AMPKα activation in *Golm1* ^pKO^ kidneys. Unexpectedly, PKA activity was unaffected in either *Golm1* ^pKO^ or *Golm1* ^pTG^ kidneys upon STZ or HFD stimulation (Figure S12C, Supporting Information). To validate the role of PPARγ, we silenced PPARγ in podocytes in mice (Figure S12D, Supporting Information). As shown in Figure 7D, PPARγ silence abolished AMPKα activation in *Golm1* ^pKO^ kidneys upon STZ injection. Accordingly, the beneficial effects of podocyte‐specific GOLM1 ablation against STZ‐induced inflammation, oxidative damage and apoptosis were significantly abolished by PPARγ silence, as evidenced by the increased TNF‐α, IL‐1β, IL‐6, MDA, 3‐NT, and 8‐OHdG levels as well as TUNEL+ cells (Figure S12E–G, Supporting Information). Meanwhile, the improved glomerulomegaly, renal fibrosis and dysfunction in *Golm1* ^pKO^ mice upon STZ treatment were also blocked by PPARγ silence (Figure S12H–J). Consistently, we also found that the anti‐inflammatory, anti‐oxidant and anti‐apoptotic effects in HFD‐treated *Golm1* ^pKO^ kidneys were abolished by PPARγ silence (Figure S13A–C, Supporting Information). Accordingly, podocyte‐specific GOLM1 ablation‐mediated protective effects against HFD‐induced glomerulomegaly, renal fibrosis and dysfunction were also blocked with sh*Pparg* infection (Figure S13D–F, Supporting Information). Moreover, we also validated the necessity of PPARγ in HG‐stimulated podocytes. Consistent with the in vivo findings, we found that PPARγ inhibition significantly abolished the resistance of *Golm1* ^pKO^ podocytes to HG‐induced cellular damage, as evidenced by the decreased cell viability and increased LDH releases (Figure S14A,B, Supporting Information). In addition, the anti‐inflammatory, and antioxidant effects in *Golm1* ^pKO^ podocytes were also blocked by GW9662 (Figure S14C–E, Supporting Information). The aforementioned data implied that GOLM1 aggravated podocyte injury and DN in an autocrine‐dependent manner, and that neutralizing extracellular GOLM1 or preventing GOLM1 cleavage dramatically prevented its DN‐promoting role. Based on these findings, we speculated that extracellular GOLM1 exerted these functions by interacting with the potential receptors on podocyte surface. As shown in Figure 7E, primary podocytes were incubated with biotin‐labeled GOLM1, and biotin‐labeled GOLM1 interacted with podocytes in a dose‐dependent and saturable manner. To determine the potential receptors of extracellular GOLM1 in podocytes, we analyzed the GOLM1‐interactome from The Human Protein Atlas database, and identified EGFR as a potential receptor of GOLM1 (Figure 7F,G). In addition, GOLM1 coimmunoprecipitated with EGFR in podocytes (Figure 7H). Next, we knocked down EGFR in podocytes to investigate whether GOLM1‐EGFR interaction was required for PPARγ inhibition in *Golm1* ^pTG^ podocytes (Figure 7I). As shown in Figure 7J,K, EGFR knockdown reduced the interaction between extracellular GOLM1 and podocytes, and PPARγ downregulation in *Golm1* ^pTG^ podocytes was also blocked by EGFR knockdown. Collectively, our results reveal that podocyte GOLM1 inactivates AMPKα pathway through interacting with EGFR to inhibit PPARγ.

### 3.8 GOLM1 Neutralizing Antibody is Sufficient to Alleviate DN in Mice

**Figure 8:**
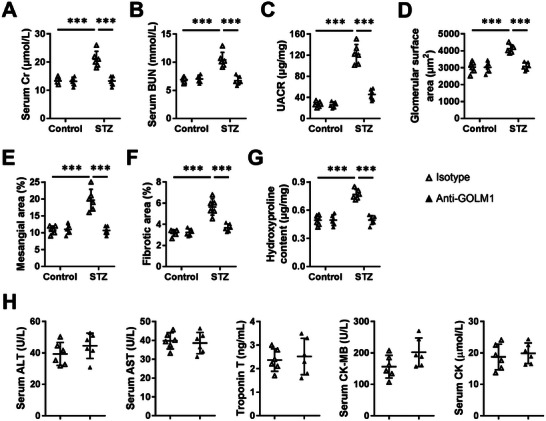
GOLM1 neutralizing antibody is sufficient to alleviate DN in mice. A,B) The levels of serum Cr and BUN. C) Quantification of UACR. D,E) Quantification of glomerular surface area and mesangial area. F) Quantification of fibrotic area. G) Quantification of renal hydroxyproline content. H) The levels of serum ALT, AST, troponin T, CK‐MB, and CK. *N* = 6 per group. *P < 0.05, **P < 0.01, ***P < 0.001 versus the matched groups.

Given the deleterious effects of podocyte GOLM1 in DN progression, we finally investigated whether GOLM1 neutralizing antibody could provide renal protection in mice. As shown in **Figure** **8**A–C, treatment with GOLM1 neutralizing antibody significantly prevented STZ‐induced renal dysfunction, as evidenced by decreased serum Cr, BUN, and UACR levels. In addition, the increased glomerulomegaly, mesangial region expansion and renal fibrosis in STZ‐treated mice were also attenuated by GOLM1 neutralizing antibody (Figure 8D–G). Mechanistically, the treatment with anti‐GOLM1 dramatically decreased the levels of podocyte‐interacted biotin‐GOLM1 in wild type podocytes, but not in EGFR‐deficient cells, indicating that anti‐GOLM1 treatment regulated DN through EGFR (Figure S15A,B, Supporting Information). Accordingly, the downstream PPARγ/AMPKα was also restored by anti‐GOLM1 treatment in diabetic kidneys (Figure S15C, Supporting Information). Of note, the treatment with anti‐GOLM1 did not result in any side effects, including hepatotoxicity, nephrotoxicity, cardiotoxicity, and muscle toxicity (Figure 8H). Collectively, our results reveal that GOLM1 neutralizing antibody is sufficient to alleviate DN in mice.

## 4 Discussion

**Figure 9:**
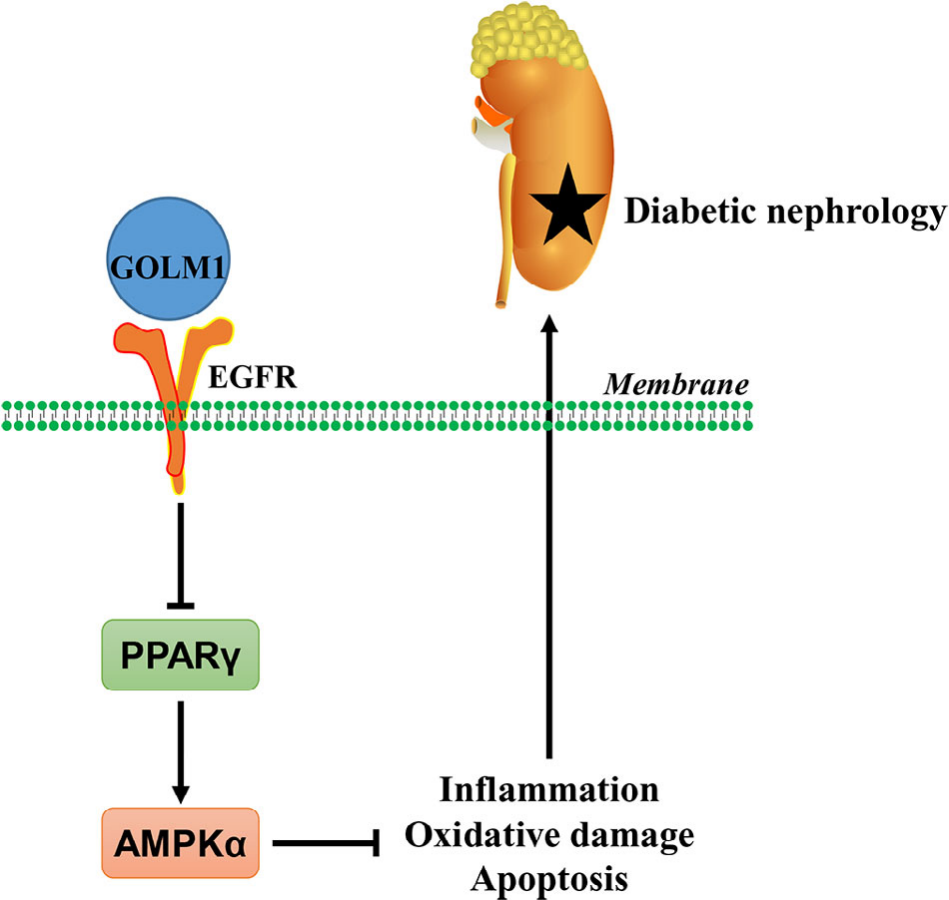
The proposed mechanisms mediating the role of GOLM1 in DN. Podocyte GOLM1 interacts with EGFR to inhibit PPARγ, and then inactivates AMPKα pathway, which facilitates diabetes‐related inflammation, oxidative damage, apoptosis, and renal dysfunction.

This study identify the crucial role of podocyte GOLM1 in the regulation of renal inflammation, oxidative damage, and dysfunction in two different DN mouse models using gain‐ and loss‐of‐function studies. Our results reveal that GOLM1 expression is elevated in diabetic kidneys and HG‐stimulated podocytes, and positively correlates with renal dysfunction during DN progression. Podocyte‐specific GOLM1 ablation prevents, while podocyte‐specific GOLM1 overexpression facilitates diabetes‐related inflammation, oxidative damage, apoptosis, and renal dysfunction in vivo and in vitro. Mechanistic studies reveal that podocyte‐specific GOLM1 ablation attenuates DN through activating AMPKα pathway, and inhibiting AMPKα pathway completely abolishes the beneficial effects in *Golm1* ^pKO^ mice or podocytes. Further findings imply that podocyte GOLM1 inactivated AMPKα pathway through interacting with epidermal growth factor receptor to inhibit PPARγ (**Figure** **9**). Moreover, treatment with GOLM1 neutralizing antibody is sufficient to alleviate DN in mice. Overall, our study for the first time identify a pathogenic role of podocyte GOLM1 in the progression of DN, and inhibiting GOLM1 represents an attractive therapeutic approach to treat DN.

The global prevalence of diabetes is significantly increased in recent years, and nearly 40% of T2DM and 30% of T1DM individuals suffer from diabetes‐induced kidney damage. However, the pathophysiological mechanisms of T1DM‐ or T2DM‐induced DN are not completely the same.^[^ ^47^, ^48^ ^]^ In T1DM, the increased glomerular basement membrane thickness and mesangial expansion are primary morphological lesions in the kidney, which result in a progressive reduction in the filtration surface of the glomerulus. However, renal lesions in T2DM are much more complex. The prevalence of nondiabetic renal lesions in proteinuric T2DM patients is high, and these patients commonly display relatively mild glomerulopathy considering the disproportionately severe changes in other renal structures, including tubular atrophy, tubular basement membrane thickening and reduplication, interstitial fibrosis, advanced glomerular arteriolar hyalinosis, and global glomerular sclerosis.^[^ ^48^ ^]^ Podocytes are reduced in both T1DM‐ and T2DM‐induced DN, and this reduction in podocytes may precede the appearance of clinically detectable proteinuria.^[^ ^49^ ^]^ So, we used both T1DM and T2DM mouse models, and focused on podocytes in our study. Hypertension is a key feature of end‐stage renal disease due to water‐sodium retention, renin‐angiotensin‐aldosterone system activation, endothelial dysfunction, insulin resistance, etc. In our study, we found that podocyte‐specific GOLM1 ablation dramatically prevented diabetes‐induced renal dysfunction and the progression of DN. Accordingly, the increased DBP and SBP in mice with diabetic nephrology were attenuated by GOLM1 deficiency. Consistent with our findings, Chen et al. demonstrated that the increased podocyte injury in podocyte‐specific SIRPα‐deficient mice aggravated renal dysfunction in diabetic mice.^[^ ^50^ ^]^ Enhancing podocyte PANoptosis also exacerbated diabetes‐induced albuminuria and kidney injury.^[^ ^51^ ^]^ DN is closely associated with renal inflammation and oxidative damage. Gao et al. determined that the proinflammatory cytokines (TNF‐α, IL‐1β, IL‐6, IL‐4) and oxidative stress markers (MDA, SOD, and GSH) were significantly elevated in diabetic kidneys or HG‐stimulated podocytes, accompanied with a severe renal dysfunction.^[^ ^52^ ^]^ NF‐κB is crucial for the induction and preservation of inflammation through triggering the transcription of proinflammatory cytokines. Fu et al. recently found that NF‐κB was activated in diabetic kidneys, and subsequently facilitated inflammatory response and renal dysfunction in mice.^[^ ^53^ ^]^ In addition, sustained high concentration blood glucose also stimulates ROS overproduction and ultimately results in oxidative damage of kidneys. Nuclear factor erythroid 2‐like 2 (NRF2) is a crucial antioxidant transcription factor, and is of high importance for the expression of antioxidant defense proteins.^[^ ^54^, ^55^ ^]^ Liu et al. recently demonstrated that *Nrf2*‐deficient mice lost the antioxidant defenses, and exhibited severe albuminuria and glomerular structural damage.^[^ ^56^ ^]^ In contrast, activation of NRF2 pathway effectively reduced oxidative damage in diabetic kidneys.^[^ ^57^ ^]^ Interestingly, feeding of EPA/DHA‐rich fish oil increased oxidative stress and activation of NRF2 in podocytes, and resulted in persistent oxidative stress adaptation, which eventually inhibited the progression of DN.^[^ ^58^ ^]^ In our study, we revealed that the elevated GOLM1 in podocytes contributed to inflammation and oxidative damage during DN progression, and that podocyte‐specific GOLM1 ablation significantly inhibited inflammation and oxidative damage in diabetic kidneys as well as HG‐stimulated podocytes.

GOLM1 is well‐known as an oncogene in many malignancies, and elevated GOLM1 expression promoted the colony formation, development, and metastasis of tumor cells.^[^ ^59^ ^]^ Emerging studies have linked GOLM1 expression with inflammation. GOLM1 expression was found to be increased in infected tissues and correlated with acute and chronic inflammatory response.^[^ ^60^, ^61^ ^]^ GOLM1 overexpression also facilitated the replication of hepatitis C virus (HCV) through inhibiting type I interferon production and subsequently prolonged HCV infection.^[^ ^62^ ^]^ In contrast, genetic inactivation of GOLM1 in myeloid cells significantly suppressed IL‐12 secretion and polarized macrophages toward M2 type.^[^ ^62^ ^]^ Meanwhile, Chen et al. also showed that GOLM1 expression was positively correlated with the regulation of an immunosuppressive environment in HCC tissues.^[^ ^63^ ^]^ Recently, Lin et al. used integrative analysis to identify the potential downstream pathways of GOLM1, and found that the DEGs downstream of GOLM1 was related to biological processes of oxidative stress and angiogenesis.^[^ ^23^ ^]^ Peng et al. also showed that the main enriched pathways downstream GOLM1 was Oxidation‐Reduction. In addition, they found that AMPK signaling pathway in the liver was significantly altered by GOLM1 overexpression.^[^ ^25^ ^]^ In our study, we revealed that GOLM1 expression was elevated in diabetic kidneys and HG‐stimulated podocytes, and positively correlated with renal dysfunction during DN progression. Podocyte‐specific GOLM1 ablation prevented, while podocyte‐specific GOLM1 overexpression facilitated diabetes‐related inflammation, oxidative damage, apoptosis, and renal dysfunction in vivo and in vitro. Further findings implied that podocyte‐specific GOLM1 ablation attenuated DN through activating AMPKα pathway. PPARγ belongs to a member of the nuclear receptor superfamily, and its elevation is essential for the activation of AMPKα.^[^ ^64^ ^]^ Zhang et al. demonstrated that PPARγ upregulation by rosmarinic acid dramatically activated AMPKα and subsequently prevented pressure overload‐induced cardiac fibrosis.^[^ ^12^ ^]^ Meanwhile, PPARγ upregulation also increases the expression and secretion of various metabolic cytokines (adipokines and myokines) to activate AMPKα. A previous study defined FGF21 as a crucial mediator linking PPARγ to the activation of AMPKα.^[^ ^65^ ^]^ In addition, Shi et al. found that PPARγ upregulation reduced oxidative stress and lipid peroxidation, and decreased UCP2 expression. Subsequently, the decrease in UCP2 levels promoted the activation of the AMPKα signaling pathway.^[^ ^66^ ^]^ Moreover, thiazolidinediones, agonists of PPARγ, have been shown to be effective in activating AMPKα to exert renal protections.^[^ ^67^ ^]^ These studies provide compelling evidences that PPARγ elevation is sufficient to activate AMPKα. Consistently, we determined that podocyte‐specific GOLM1 ablation activated AMPKα to prevent DN through upregulating PPARγ. Further findings indicated that extracellular GOLM1 directly bound to EGFR to inhibit PPARγ expression. Li et al. revealed that high glucose dramatically increased the activation of EGFR signaling pathway in podocytes, and subsequently contributes to podocyte injury and progression of DN.^[^ ^68^ ^]^ Accordingly, the inhibition of EGFR activation is associated with improved renal function in diabetic mice.^[^ ^69^ ^]^ EGFR acts as a receptor on the cell membrane and is an upstream activators of both AKT/GSK3β/β‐Catenin and MEK1/2‐ERK1/2 signaling pathways.^[^ ^70^ ^]^ It is well‐accepted that EGFR activation increased AKT/GSK3β phosphorylation, resulting in GSK3β inactivation, and that GSK3β inactivation prevented β‐Catenin degradation and promoted its nuclear accumulation.^[^ ^71^ ^]^ Findings from Reggio et al. and Qian et al. implied that β‐catenin stabilization directly limited PPARγ expression.^[^ ^72^, ^73^ ^]^ In addition, enhancing AKT/GSK3β phosphorylation also elevated Snail expression, which suppressed the expression of PPARγ by directly binding to the E‐box in the PPARγ gene promoter.^[^ ^74^ ^]^ Moreover, the activation of MEK1/2‐ERK1/2 signaling pathway led to the nuclear export and downregulation of PPARγ by physiological interaction, eventually repressing its transcriptional activity.^[^ ^75^ ^]^ Accordingly, our study implies that podocyte GOLM1 inactivates AMPKα pathway through interacting with EGFR to inhibit PPARγ.

Overall, our study for the first time identify a pathogenic role of podocyte GOLM1 in the progression of DN, and inhibiting GOLM1 represents an attractive therapeutic approach to treat DN.

## Conflict of Interest

The authors declare no conflict of interest.

## Supporting information



Supporting Information

## Data Availability

The data that support the findings of this study are available on request from the corresponding author. The data are not publicly available due to privacy or ethical restrictions.
