# Supplementary material for: Inhibition of AMPKα Pathway by Podocyte GOLM1 Exacerbates Diabetic Nephrology in Mice
Source: Adv Sci (Weinh). 2025 Jul 13;12(37):e05695. doi: 10.1002/advs.202505695 (PMC12499477; doi:10.1002/advs.202505695)
Supplement: Supplementary file 1 — Supporting Information [file ADVS-12-e05695-s001.docx]

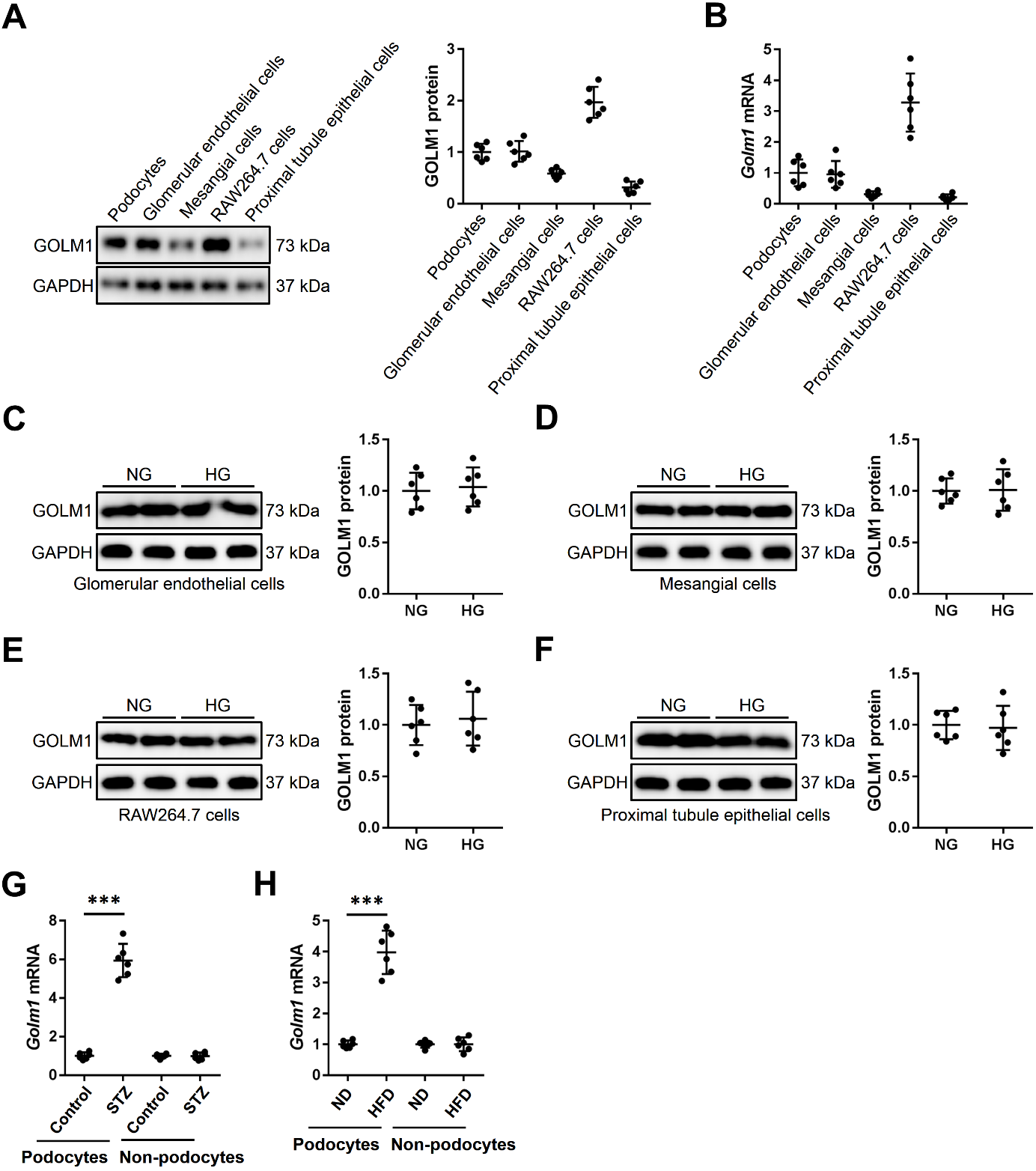


Figure S1. The expression and alteration of GOLM1 in different kidney cells. (A-B) GOLM1 mRNA and protein levels in different kidney cells. (C) GOLM1 protein level in glomerular endothelial cells with or without HG stimuli. (D) GOLM1 protein level in mesangial cells with or without HG stimuli. (E) GOLM1 protein level in RAW264.7 cells with or without HG stimuli. (F) GOLM1 protein level in proximal tubule epithelial cells with or without HG stimuli. (G-H) Podocytes and non-podocytes were isolated from STZ- or HFD-induced diabetic kidneys, and then subjected to detect GOLM1 mRNA levels. *N* = 6 per group. **P* < 0.05, ***P* < 0.01, ****P* < 0.001 versus the matched groups. NS indicates no significance.


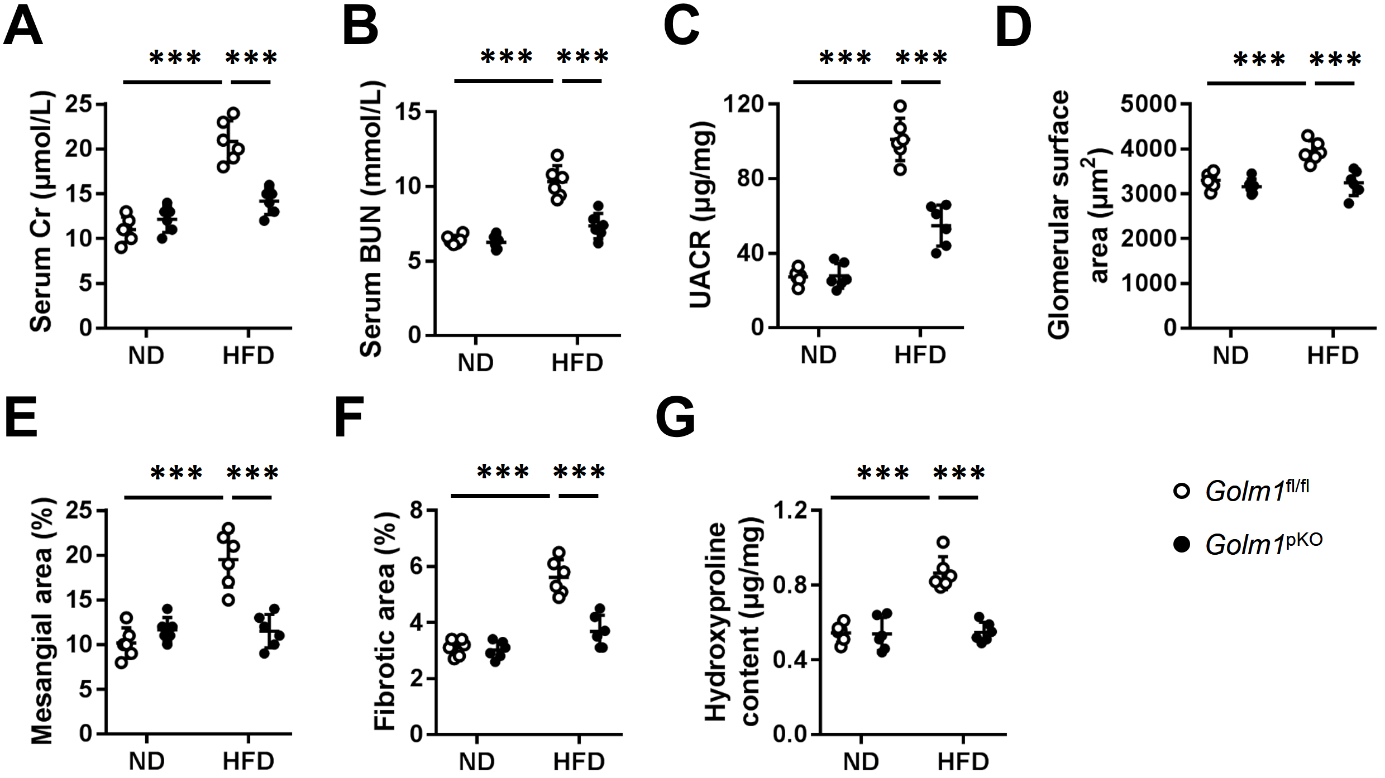


Figure S2. Podocyte-specific GOLM1 ablation prevents DN in HFD-treated mice. (A-B) The levels of serum Cr and BUN. (C) Quantification of UACR. (D-E) Quantification of glomerular surface area and mesangial area. (F) Quantification of fibrotic area. (G) Quantification of renal hydroxyproline content. *N* = 6 per group. **P* < 0.05, ***P* < 0.01, ****P* < 0.001 versus the matched groups.


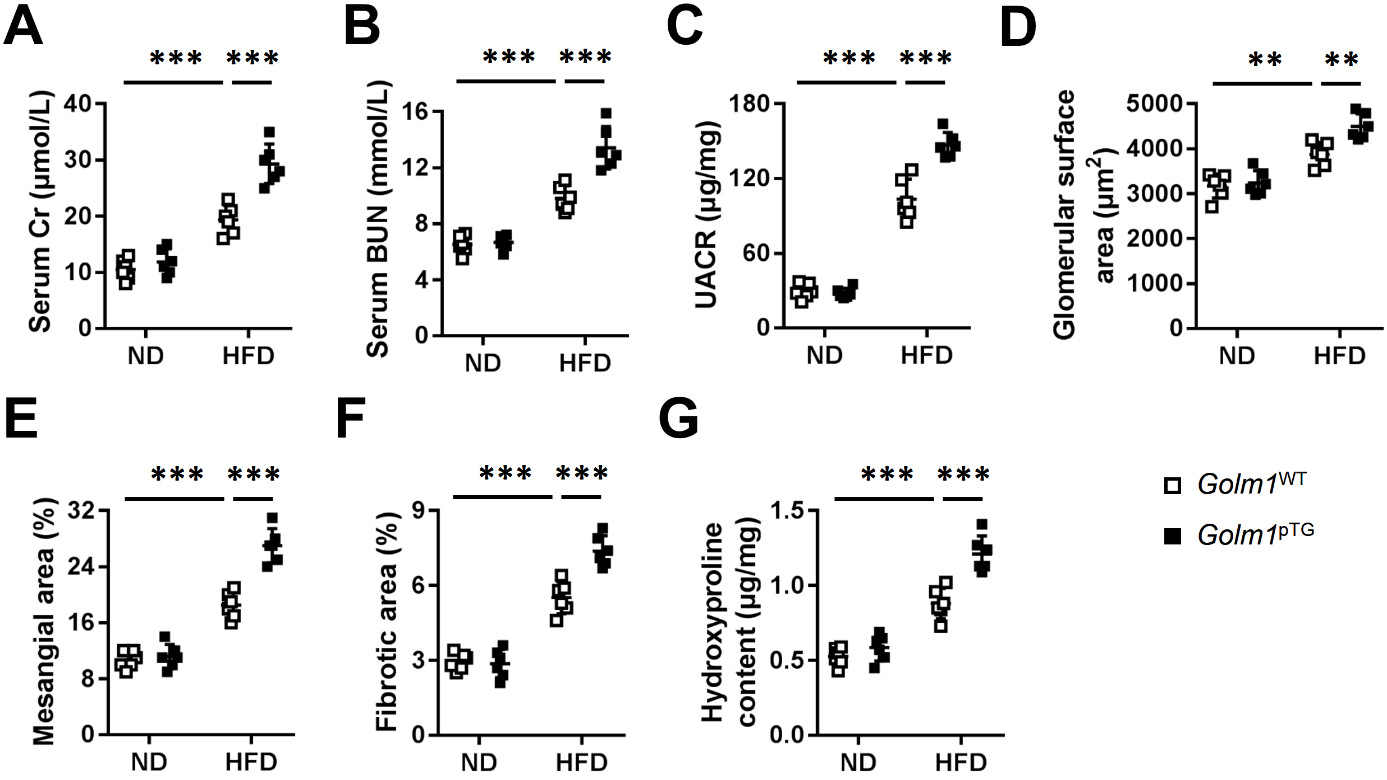


Figure S3. Podocyte-specific GOLM1 overexpression facilitates DN in HFD-treated mice. (A-B) The levels of serum Cr and BUN. (C) Quantification of UACR. (D-E) Quantification of glomerular surface area and mesangial area. (F) Quantification of fibrotic area. (G) Quantification of renal hydroxyproline content. *N* = 6 per group. **P* < 0.05, ***P* < 0.01, ****P* < 0.001 versus the matched groups.


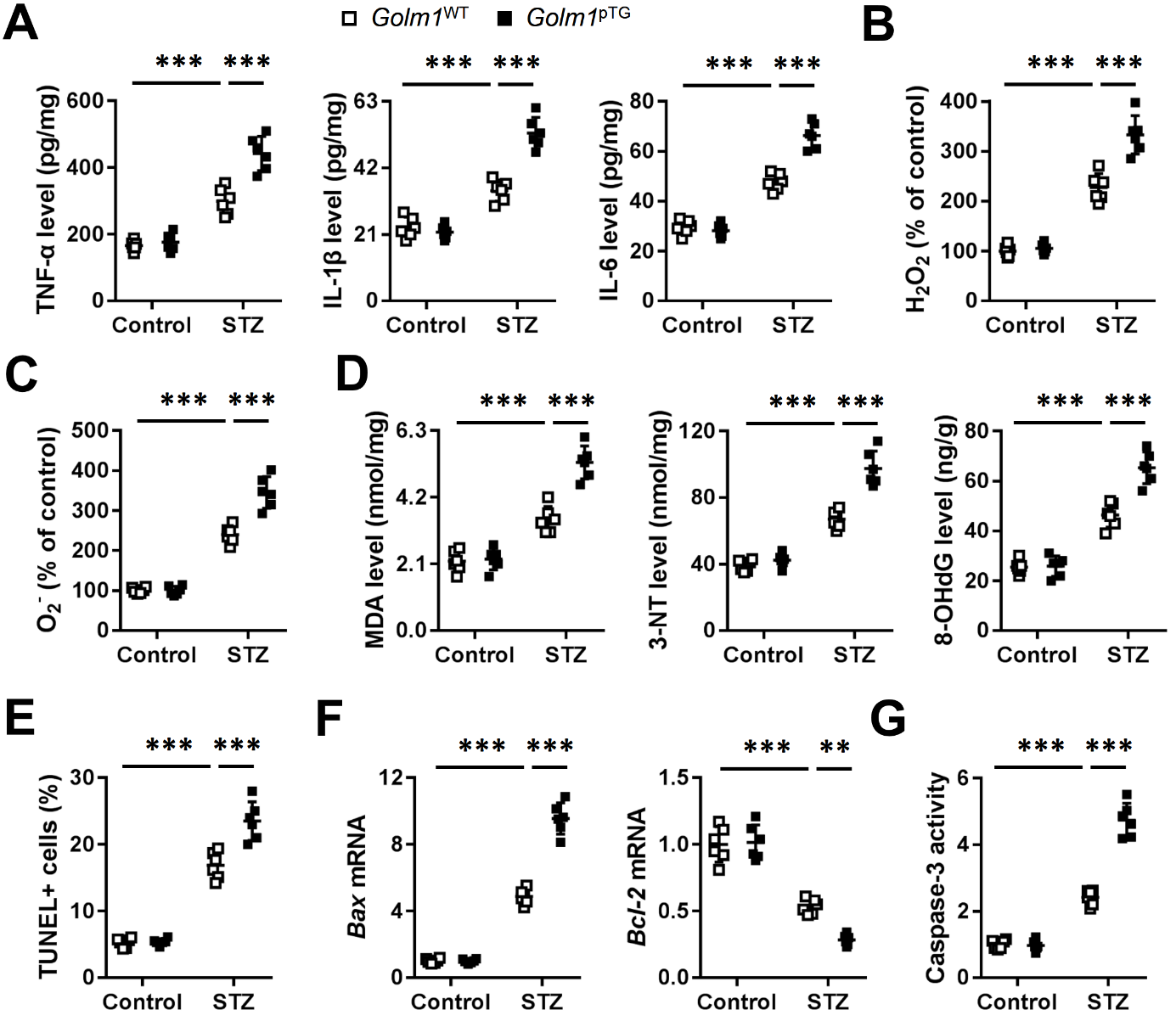


Figure S4. Podocyte-specific GOLM1 overexpression promotes inflammation and oxidative damage in STZ-treated mice. (A) The levels of renal TNF-α, IL-1β and IL-6. (B-C) The levels of H_2_O_2_ and O_2_^-^ in kidneys. (D) The levels of renal MDA, 3-NT and 8-OHdG. (E) Quantification of TUNEL+ cells. (F) Relative *Bax* and *Bcl-2* mRNA levels in kidneys. (G) Relative caspase-3 activity in kidneys. *N* = 6 per group. **P* < 0.05, ***P* < 0.01, ****P* < 0.001 versus the matched groups.


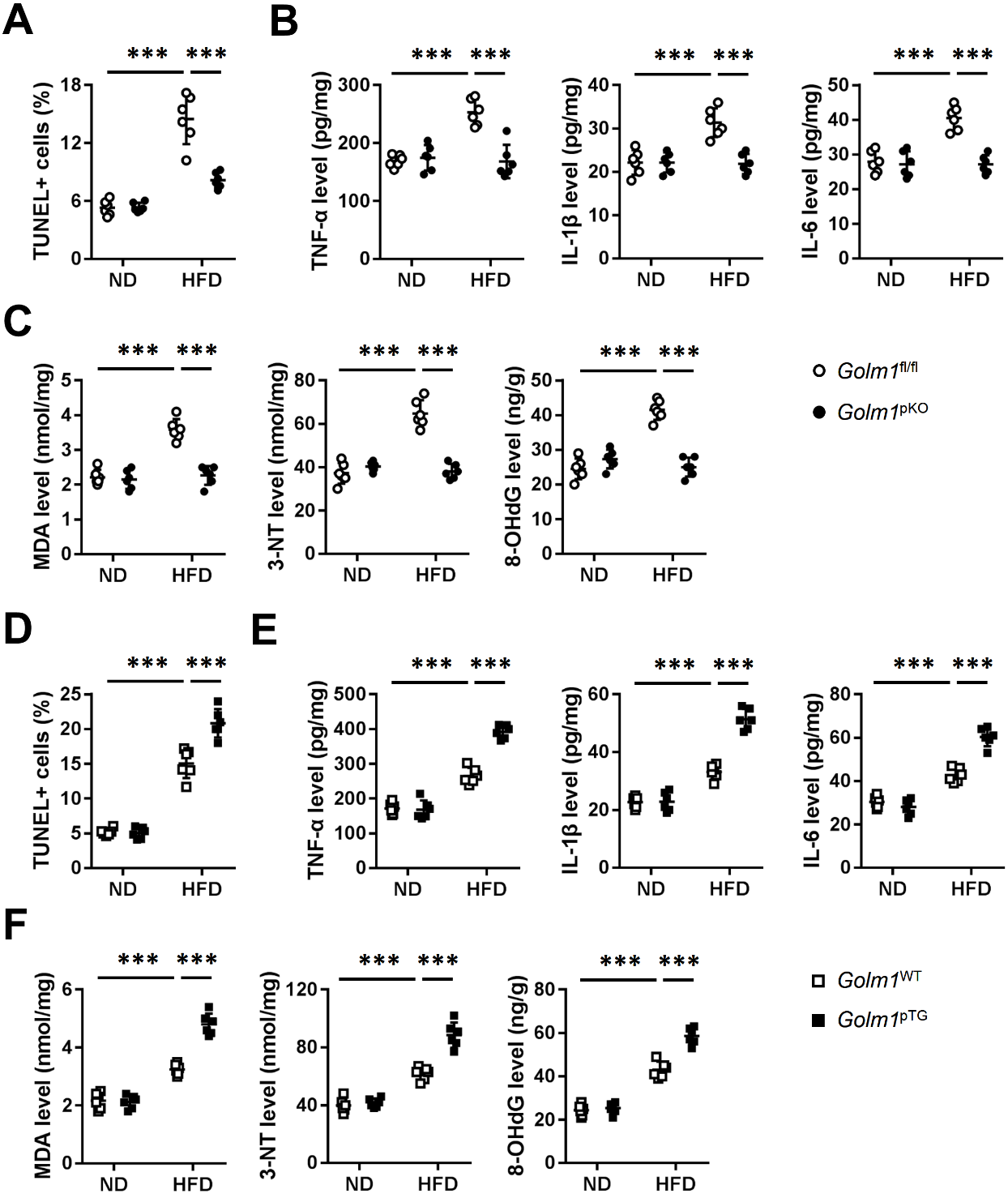


Figure S5. Podocyte-specific GOLM1 ablation decreases, while podocyte-specific GOLM1 overexpression increases inflammation and oxidative damage in HFD-treated mice. (A) Quantification of TUNEL+ cells in kidneys from *Golm1*^pKO^ or *Golm1*^fl/fl^ mice. (B) The levels of renal TNF-α, IL-1β and IL-6 in *Golm1*^pKO^ or *Golm1*^fl/fl^ mice. (C) The levels of renal MDA, 3-NT and 8-OHdG in *Golm1*^pKO^ or *Golm1*^fl/fl^ mice. (D) Quantification of TUNEL+ cells in kidneys from *Golm1*^pTG^ or *Golm1*^WT^ mice. (E) The levels of renal TNF-α, IL-1β and IL-6 in *Golm1*^pTG^ or *Golm1*^WT^ mice. (F) The levels of renal MDA, 3-NT and 8-OHdG in *Golm1*^pTG^ or *Golm1*^WT^ mice. *N* = 6 per group. **P* < 0.05, ***P* < 0.01, ****P* < 0.001 versus the matched groups.


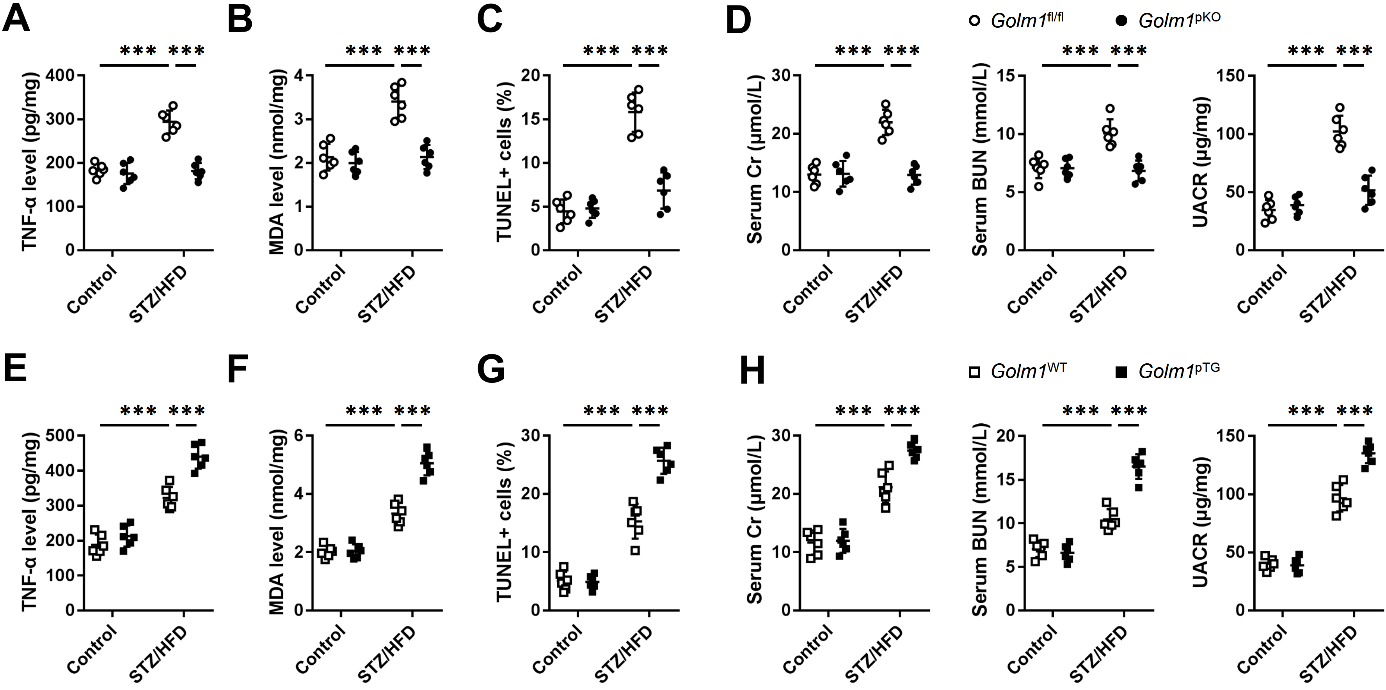


Figure S6. Podocyte-specific GOLM1 ablation decreases, while podocyte-specific GOLM1 overexpression increases inflammation, oxidative damage, apoptosis and renal dysfunction in STZ/HFD-treated mice. (A) The levels of renal TNF-α in *Golm1*^pKO^ or *Golm1*^fl/fl^ mice. (B) The levels of renal MDA in *Golm1*^pKO^ or *Golm1*^fl/fl^ mice. (C) Quantification of TUNEL+ cells in kidneys from *Golm1*^pKO^ or *Golm1*^fl/fl^ mice. (D) The levels of serum Cr, BUN and UACR from *Golm1*^pKO^ or *Golm1*^fl/fl^ mice. (E) The levels of renal TNF-α in *Golm1*^pTG^ or *Golm1*^WT^ mice. (F) The levels of renal MDA in *Golm1*^pTG^ or *Golm1*^WT^ mice. (G) Quantification of TUNEL+ cells in kidneys from *Golm1*^pTG^ or *Golm1*^WT^ mice. (H) The levels of serum Cr, BUN and UACR from *Golm1*^pTG^ or *Golm1*^WT^ mice. *N* = 6 per group. **P* < 0.05, ***P* < 0.01, ****P* < 0.001 versus the matched groups.


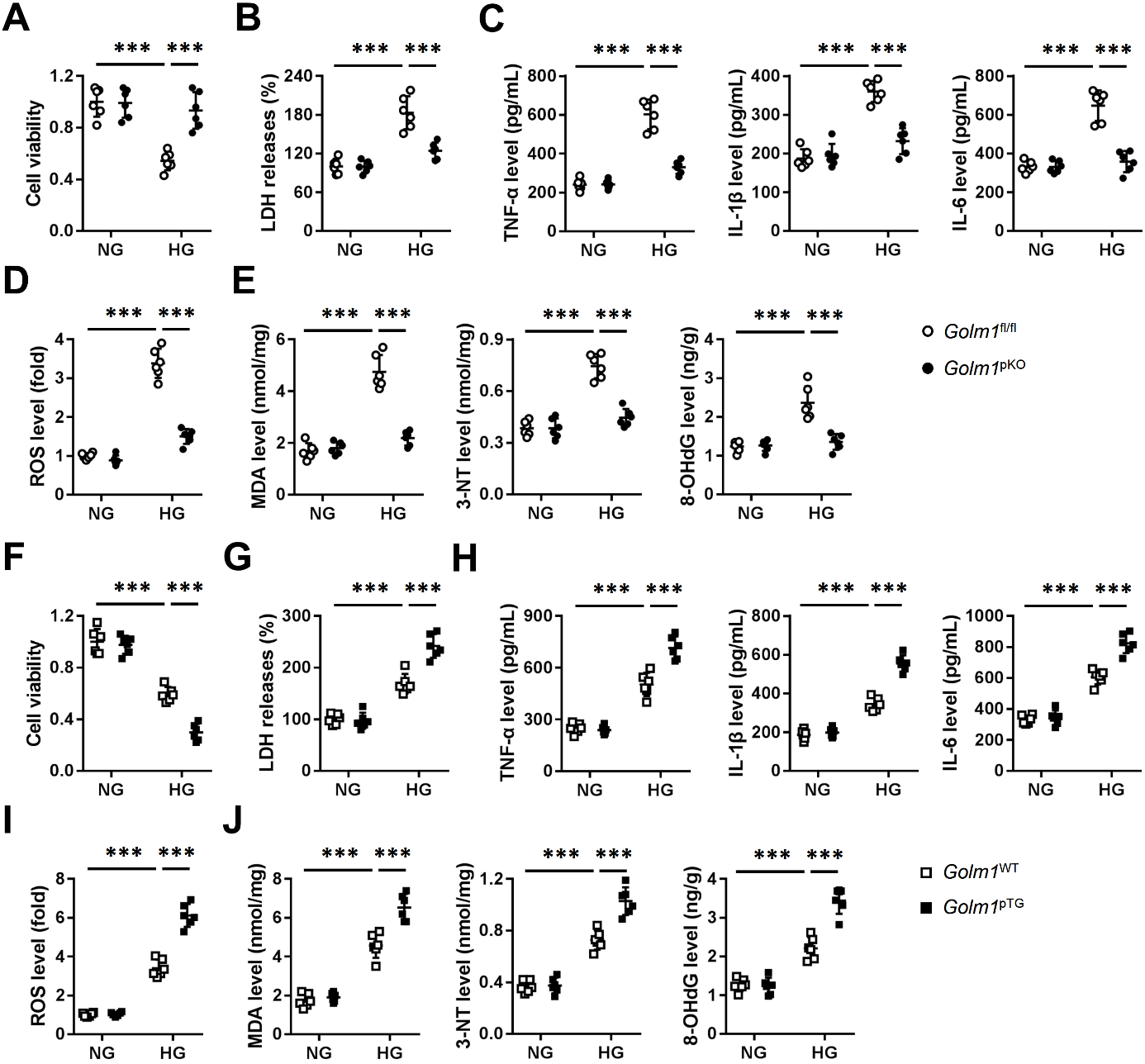


Figure S7. Podocyte GOLM1 aggravates high glucose-induced inflammation and oxidative damage in vitro. (A) Cell viability in *Golm1*^pKO^ or *Golm1*^fl/fl^ podocytes. (B) LDH releases in *Golm1*^pKO^ or *Golm1*^fl/fl^ podocytes. (C) The levels of TNF-α, IL-1β and IL-6 in the medium from *Golm1*^pKO^ or *Golm1*^fl/fl^ podocytes. (D) Quantification of ROS level in *Golm1*^pKO^ or *Golm1*^fl/fl^ podocytes. (E) The levels of MDA, 3-NT and 8-OHdG in *Golm1*^pKO^ or *Golm1*^fl/fl^ podocytes. (F) Cell viability in *Golm1*^pTG^ or *Golm1*^WT^ podocytes. (G) LDH releases in *Golm1*^pTG^ or *Golm1*^WT^ podocytes. (H) The levels of TNF-α, IL-1β and IL-6 in the medium from *Golm1*^pTG^ or *Golm1*^WT^ podocytes. (I) Quantification of ROS level in *Golm1*^pTG^ or *Golm1*^WT^ podocytes. (J) The levels of MDA, 3-NT and 8-OHdG in *Golm1*^pTG^ or *Golm1*^WT^ podocytes. *N* = 6 per group. **P* < 0.05, ***P* < 0.01, ****P* < 0.001 versus the matched groups.


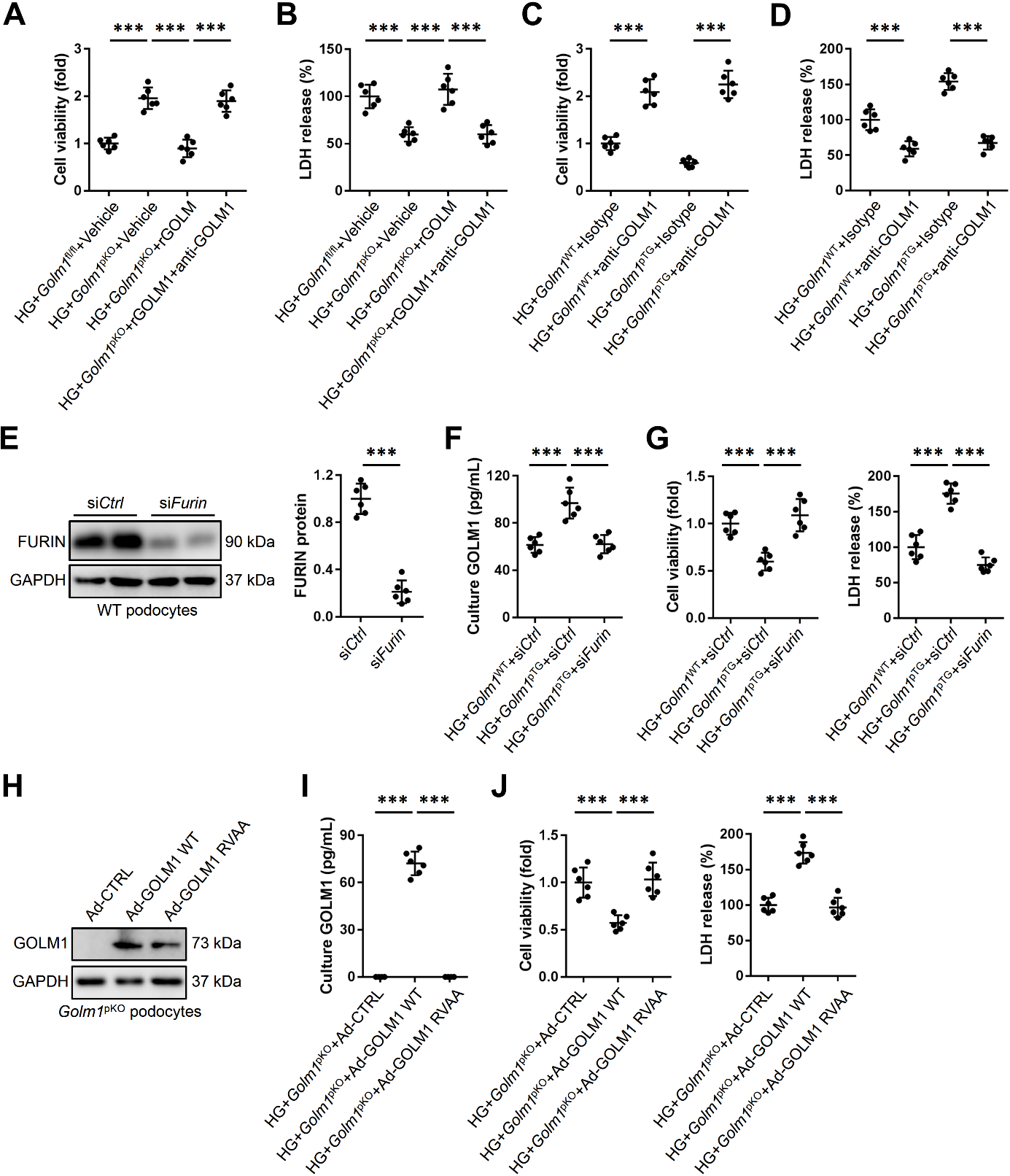


Figure S8. Podocyte GOLM1 aggravates high glucose-induced inflammation and oxidative damage in an autocrine-dependent manner in vitro. (A-B) HG-stimulated *Golm1*^pKO^ podocytes were treated with rGOLM1 in the presence or absence of anti-GOLM1, and cell viability as well as LDH releases were determined. (C-D) HG-stimulated *Golm1*^WT^ and *Golm1*^pTG^ podocytes were treated with anti-GOLM1, and cell viability as well as LDH releases were determined. (E) FURIN protein levels in podocytes with or without FURIN knockdown. (F) GOLM1 level in the medium from *Golm1*^pTG^ podocytes with or without FURIN knockdown. (G) Cell viability and LDH releases in *Golm1*^pTG^ podocytes with or without FURIN knockdown. (H) GOLM1 protein levels in *Golm1*^pKO^ podocytes with GOLM1 WT or GOLM1 RVAA overexpression. (I) GOLM1 level in the medium from *Golm1*^pKO^ podocytes with GOLM1 WT or GOLM1 RVAA overexpression. (J) Cell viability and LDH releases in *Golm1*^pKO^ podocytes with GOLM1 WT or GOLM1 RVAA overexpression. *N* = 6 per group. **P* < 0.05, ***P* < 0.01, ****P* < 0.001 versus the matched groups.


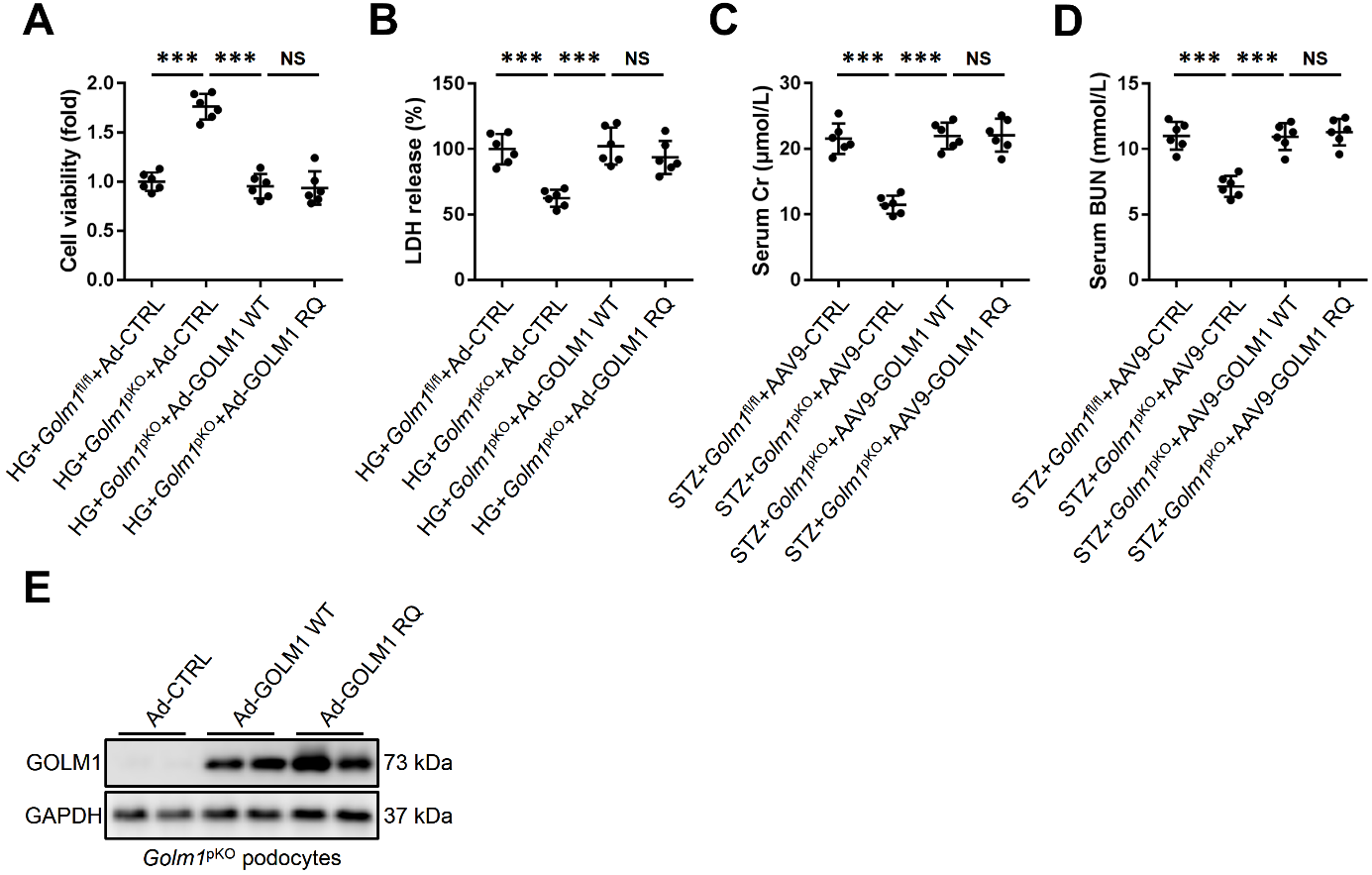


Figure S9. Podocyte GOLM1 aggravates DN in a GAP-independent manner. (A-B) Cell viability and LDH releases in *Golm1*^pKO^ podocytes with GOLM1 WT or GOLM1 RQ overexpression. (C-D) Serum Cr and BUN levels in *Golm1*^pKO^ mice with GOLM1 WT or GOLM1 RQ overexpression. (E) GOLM1 protein levels in *Golm1*^pKO^ podocytes with GOLM1 WT or GOLM1 RQ overexpression. *N* = 6 per group. **P* < 0.05, ***P* < 0.01, ****P* < 0.001 versus the matched groups.


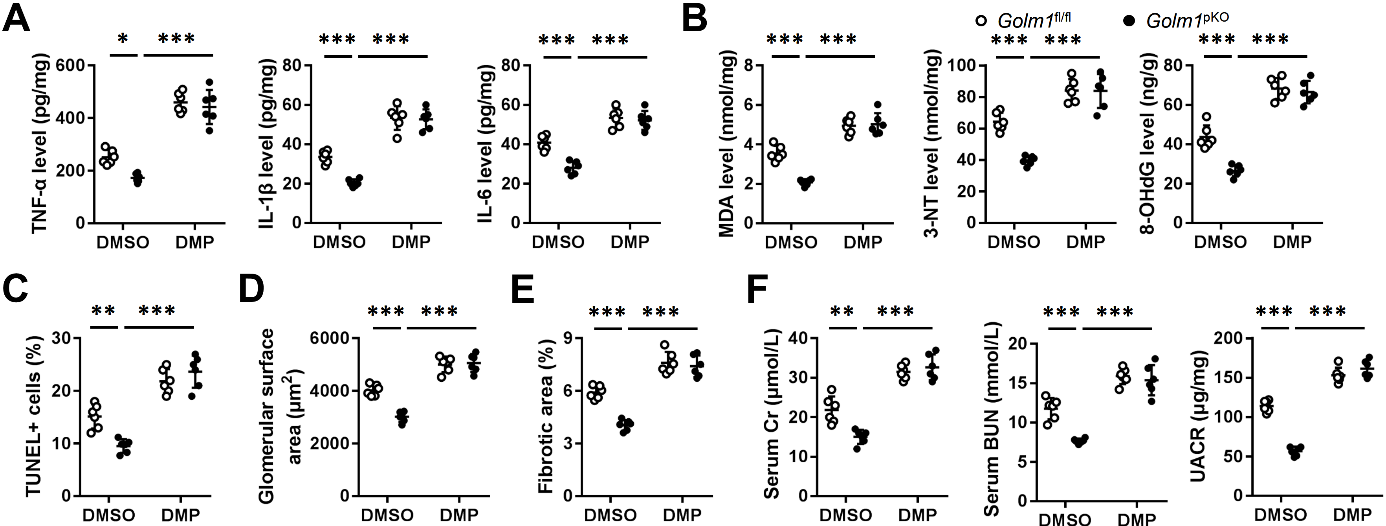


Figure S10. Podocyte-specific GOLM1 ablation attenuates HFD-induced DN through activating AMPKα pathway. (A) The levels of renal TNF-α, IL-1β and IL-6. (B) The levels of renal MDA, 3-NT and 8-OHdG. (C) Quantification of TUNEL+ cells. (D-E) Quantification of glomerular surface area and fibrotic area. (F) The levels of serum Cr, BUN and quantification of UACR. *N* = 6 per group. **P* < 0.05, ***P* < 0.01, ****P* < 0.001 versus the matched groups.


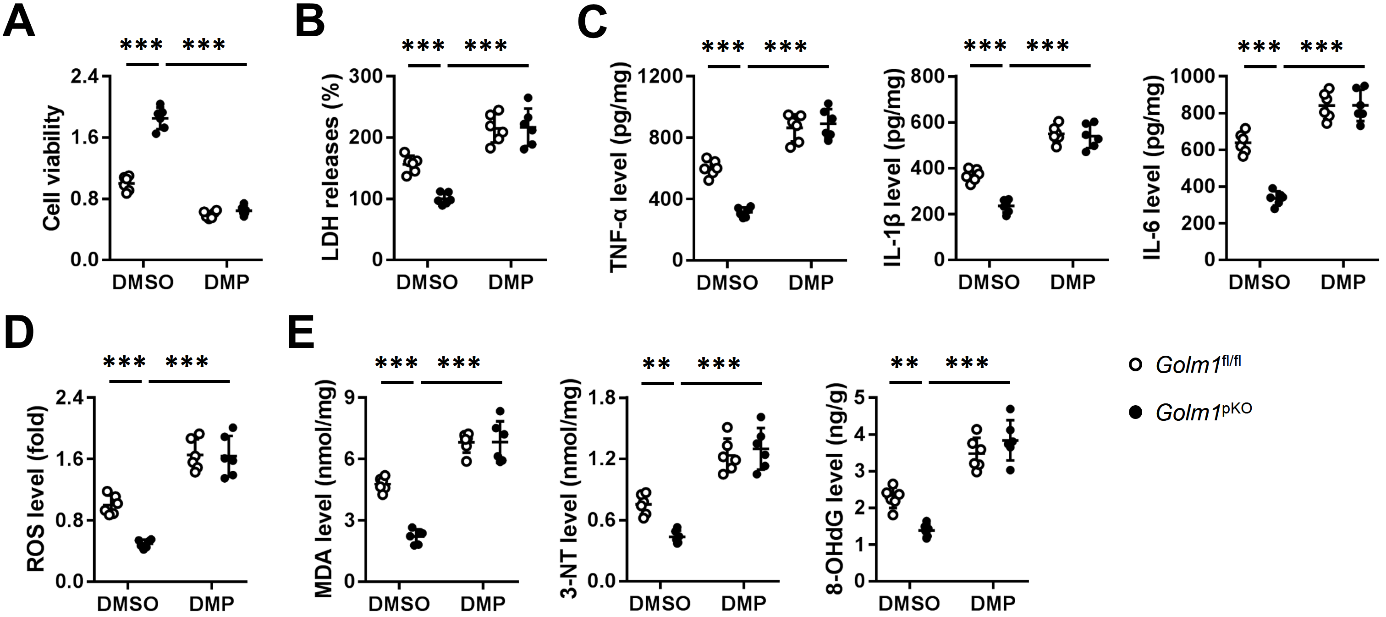


Figure S11. GOLM1 knockout reduces HG-induced inflammation and oxidative damage in podocytes through activating AMPKα pathway. (A) Cell viability in HG-stimulated *Golm1*^pKO^ or *Golm1*^fl/fl^ podocytes with or without AMPKα inhibition. (B) LDH releases in HG-stimulated *Golm1*^pKO^ or *Golm1*^fl/fl^ podocytes with or without AMPKα inhibition. (C) The levels of TNF-α, IL-1β and IL-6 in the medium from HG-stimulated *Golm1*^pKO^ or *Golm1*^fl/fl^ podocytes with or without AMPKα inhibition. (D) Quantification of ROS level in HG-stimulated *Golm1*^pKO^ or *Golm1*^fl/fl^ podocytes with or without AMPKα inhibition. (E) The levels of MDA, 3-NT and 8-OHdG in HG-stimulated *Golm1*^pKO^ or *Golm1*^fl/fl^ podocytes with or without AMPKα inhibition. *N* = 6 per group. **P* < 0.05, ***P* < 0.01, ****P* < 0.001 versus the matched groups.


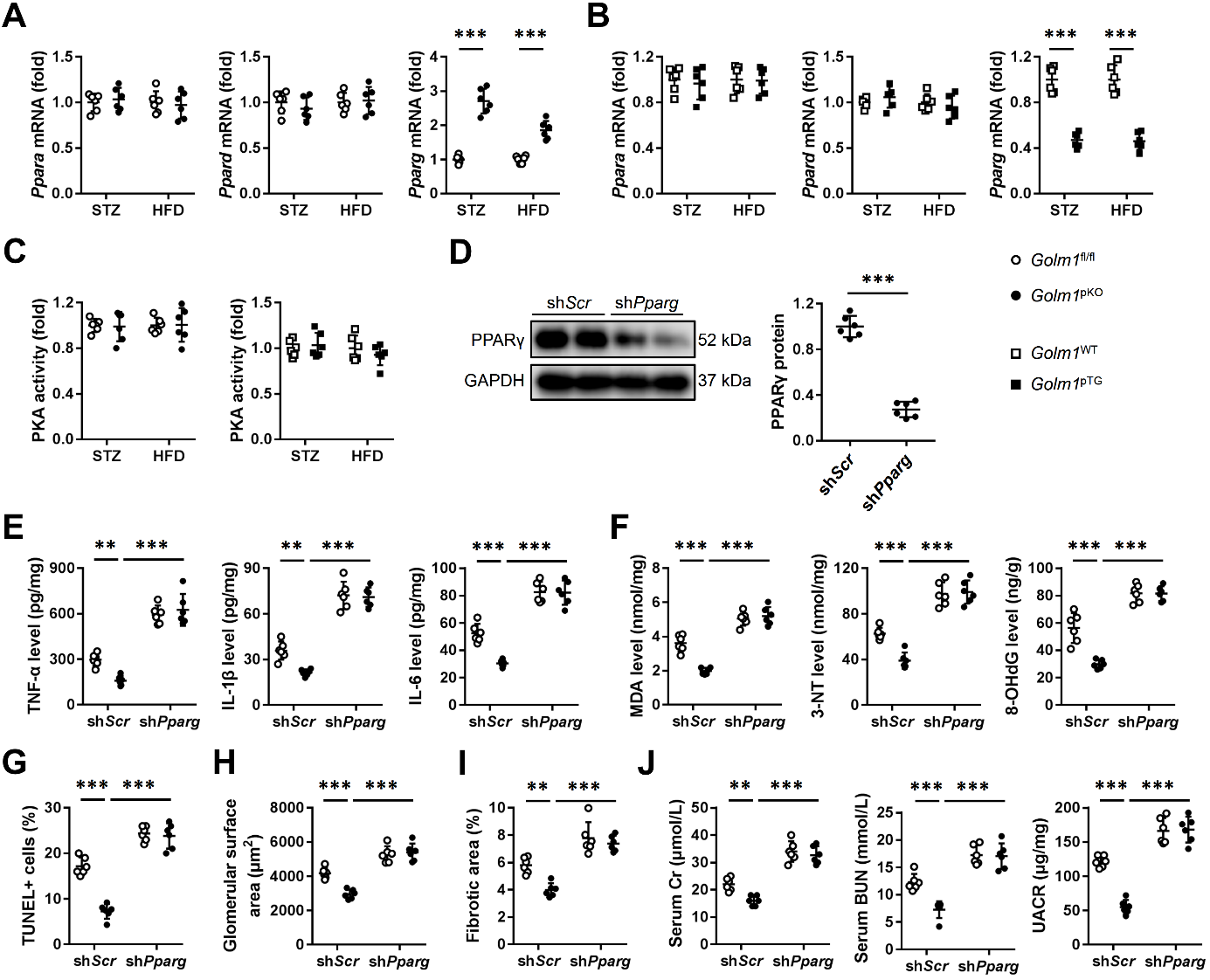


Figure S12. The effect of podocyte GOLM1 on PPARs expression and PKA activity. (A) Relative *Ppara*, *Ppard* and *Pparg* mRNA levels in *Golm1*^pKO^ or *Golm1*^fl/fl^ kidneys with STZ or HFD treatment. (B) Relative *Ppara*, *Ppard* and *Pparg* mRNA levels in *Golm1*^pTG^ or *Golm1*^WT^ kidneys with STZ or HFD treatment. (C) Relative PKA activities in *Golm1*^pKO^, *Golm1*^pTG^ or matched control kidneys with STZ or HFD treatment. (D) PPARγ protein levels in kidneys with sh*Pparg* or sh*Scr* injection. (E) The levels of renal TNF-α, IL-1β and IL-6. (F) The levels of renal MDA, 3-NT and 8-OHdG. (G) Quantification of TUNEL+ cells. (H-I) Quantification of glomerular surface area and fibrotic area. (J) The levels of serum Cr, BUN and quantification of UACR. *N* = 6 per group. **P* < 0.05, ***P* < 0.01, ****P* < 0.001 versus the matched groups.


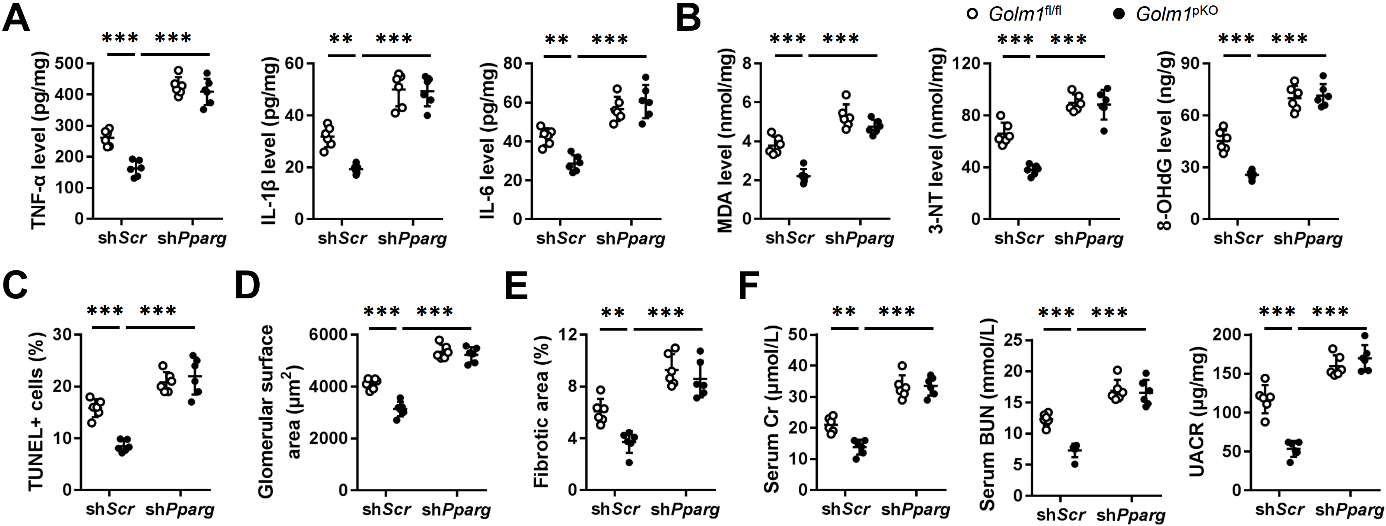


Figure S13. Podocyte-specific GOLM1 ablation attenuates HFD-induced DN through upregulating PPARγ. (A) The levels of renal TNF-α, IL-1β and IL-6. (B) The levels of renal MDA, 3-NT and 8-OHdG. (C) Quantification of TUNEL+ cells. (D-E) Quantification of glomerular surface area and fibrotic area. (F) The levels of serum Cr, BUN and quantification of UACR. *N* = 6 per group. **P* < 0.05, ***P* < 0.01, ****P* < 0.001 versus the matched groups.


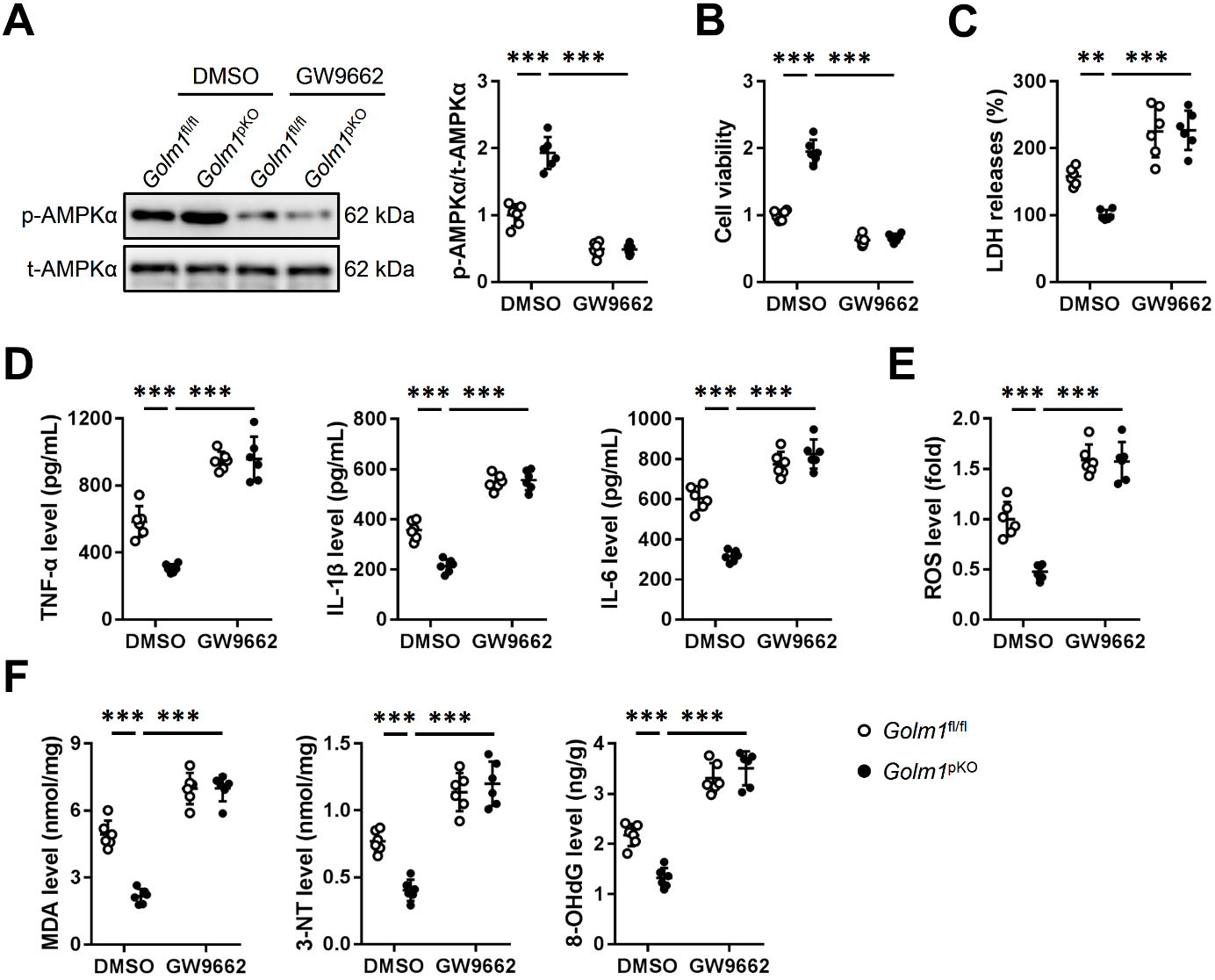


Figure S14. Podocyte GOLM1 ablation suppresses high glucose-induced inflammation and oxidative damage through upregulating PPARγ in vitro. (A) The levels of AMPKα phosphorylation in HG-stimulated *Golm1*^pKO^ or *Golm1*^fl/fl^ podocytes with or without PPARγ inhibition. (B) Cell viability in HG-stimulated *Golm1*^pKO^ or *Golm1*^fl/fl^ podocytes with or without PPARγ inhibition. (C) LDH releases in HG-stimulated *Golm1*^pKO^ or *Golm1*^fl/fl^ podocytes with or without PPARγ inhibition. (D) The levels of TNF-α, IL-1β and IL-6 in the medium from HG-stimulated *Golm1*^pKO^ or *Golm1*^fl/fl^ podocytes with or without PPARγ inhibition. (E) Quantification of ROS level in HG-stimulated *Golm1*^pKO^ or *Golm1*^fl/fl^ podocytes with or without PPARγ inhibition. (F) The levels of MDA, 3-NT and 8-OHdG in HG-stimulated *Golm1*^pKO^ or *Golm1*^fl/fl^ podocytes with or without PPARγ inhibition. *N* = 6 per group. **P* < 0.05, ***P* < 0.01, ****P* < 0.001 versus the matched groups.


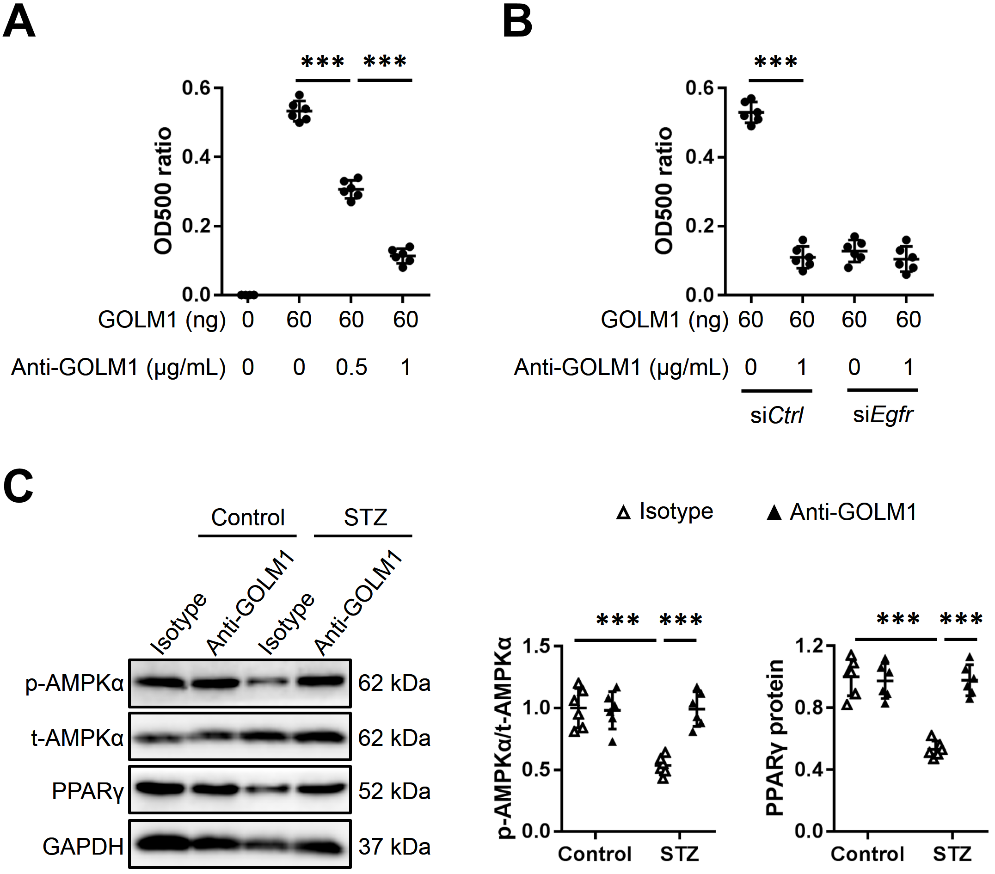


Figure S15. GOLM1 neutralizing antibody is sufficient to alleviate DN in mice. (A) Podocytes were incubated with biotin-labeled GOLM1 in the presence or absence of anti-GOLM1, and podocytes-interacted biotin-GOLM1 was determined by a colorimetric assay. (B) Wild type or EGFR-deficient podocytes were incubated with biotin-labeled GOLM1 in the presence or absence of anti-GOLM1, and podocytes-interacted biotin-GOLM1 was determined by a colorimetric assay. (C) AMPKα phosphorylation and PPARγ protein levels in STZ-treated diabetic kidneys with or without anti-GOLM1 treatment. *N* = 6 per group. **P* < 0.05, ***P* < 0.01, ****P* < 0.001 versus the matched groups.
